# Inhibition of high risk HPV31 E8^E2 repressor activity enables differentiation-independent genome amplification and E4 expression

**DOI:** 10.1371/journal.ppat.1014330

**Published:** 2026-06-08

**Authors:** Tina Melanie Rehm, Elke Straub, John Doorbar, Adam Grundhoff, Thomas Günther, Patrick Blümke, Thomas Iftner, Frank Stubenrauch

**Affiliations:** 1 Institute for Medical Virology and Epidemiology of Viral Diseases, University of Tuebingen, Tuebingen, Germany; 2 Department of Pathology, University of Cambridge, Cambridge, United Kingdom; 3 Leibniz Institute of Virology, Hamburg, Germany; University of North Carolina at Chapel Hill, UNITED STATES OF AMERICA

## Abstract

Persistent infections with high-risk human papillomaviruses (HPV) can result in different malignancies. Productive replication of HPV is normally restricted to suprabasal keratinocytes that have entered terminal differentiation and is characterized by vegetative genome amplification, activation of the late promoter, and expression of the viral late E4 protein. Cells undergoing productive replication remain in a prolonged G2 phase and exit the cell cycle without division. The viral E8^E2 protein binds to NCoR/SMRT co-repressor complexes to repress viral transcription and replication in undifferentiated keratinocytes, but the biological rationale for this repression has remained unclear. Recent studies have revealed that Mus musculus PV1 E8^E2 prevents late viral E4 expression in undifferentiated cells to enable tumor formation in vivo. Here, we demonstrate that loss of E8^E2 function in high-risk HPV31 leads to inappropriate activation of genome amplification in undifferentiated keratinocytes, resulting in expression of E4 protein and cell cycle perturbation which explains why HPV31 E8^E2 mutant genomes fail to be maintained as episomes and instead are always found integrated in surviving cell lines. Interestingly, this is independent from E4 expression suggesting that vegetative genome amplification is sufficient to prevent cell division. Remarkably, depletion of NCoR/SMRT complexes in cell lines maintaining HPV31 episomes phenocopies E8^E2 inactivation and induces genome amplification and E4 expression. Notably, most E4-positive cells generated by E8^E2 inactivation or NCoR/SMRT depletion retain basal-like characteristics, indicating that genome amplification and E4 expression can be uncoupled from differentiation when E8^E2 repression is relieved. Interestingly, differentiation diminishes the effects of NCoR/SMRT depletion, suggesting that E8^E2 activity is likely inactivated during differentiation to permit productive replication. Collectively, these findings identify E8^E2 as a critical gatekeeper preventing premature genome amplification and E4 expression in basal keratinocytes and suggest that targeting the E8^E2–NCoR/SMRT interaction may represent a novel antiviral strategy.

## Author summary

Human papillomaviruses (HPVs) are common viruses that can cause cancer by establishing long-term infections in the skin and mucosal tissues. To persist, HPV carefully controls when it replicates its DNA and produces new virus particles. Normally, productive virus replication occurs only in cells that have begun to differentiate, while infected basal cells continue to divide and maintain the viral genome. In this study, we show that this process is actively regulated by the viral E8^E2 protein. When E8^E2 is inactivated in high-risk HPV31, the virus prematurely enters its productive replication cycle in undifferentiated basal-like cells, preventing stable long-term maintenance of the viral genome. We also show that removing cellular NCoR/SMRT protein complexes, which are required for E8^E2 function, triggers genome amplification and E4 expression in undifferentiated cells. In contrast, loss of NCoR/SMRT has no additional effect in differentiated cells, indicating that cell differentiation itself suppresses E8^E2 activity. Together, our findings explain how HPV prevents premature virus production to maintain persistent infection and highlight a potential antiviral strategy that could eliminate infected cells by forcing them into the productive replication cycle.

## Introduction

Persistent infections with high risk human papillomaviruses (HPV) can result in anogenital and oropharyngeal cancers [1]. HPV have adapted their replication cycle to the differentiation state of the infected keratinocytes [2–4]. Upon infection of undifferentiated keratinocytes in the basal layer, only limited genome replication takes place and viral gene expression is restricted to the early region. The productive cycle begins when infected cells move into the suprabasal layer and start their terminal differentiation program. The first step is an re-entry into the cell cycle. In the second phase, viral genomes undergo vegetative amplification in the G2-phase of the cell cycle and this coincides with the activation of the viral late promoter (PL). PL drives the expression of the abundant *E1^E4*, *E5* transcript which results in the high-level expression of the non-structural, cytoplasmic viral E4 protein. In the third phase, terminally-differentiated, infected cells eventually exit the cell cycle and the viral L1 and L2 capsid proteins are expressed from PL transcripts that are polyadenylated at the late, and not at the early, polyA site. The differentiation-dependency of productive HPV replication has been partially ascribed to the transcriptional activation of PL by differentiation-regulated host cell transcription factors such as the activator form of C/EBP-beta, since PL can also drive the expression of the viral E1 and E2 replication activator proteins [5–8]. In addition, the activation of the ATM and ATR DNA damage response pathways by HPV is required for the differentiation-dependent vegetative genome amplification [9,10]. This might be related to the observation that HPV genomes are replicated in differentiated keratinocytes not only bi-directionally via theta type replication intermediates, but also unidirectionally presumably by a recombination-dependent replication mechanism [11,12].

The viral DNA-binding E2 protein is not only required for the recruitment of E1 to initiate replication of the viral genome, but also acts as a transcriptional modulator of viral gene expression and enables viral genome segregation in dividing cells [13,14]. In addition to E2, papillomaviruses express the conserved E8^E2 protein from the spliced *E8^E2* transcript. E8^E2 retains the non-conserved hinge region and conserved C-terminal DNA binding and dimerization domain of E2, but the conserved N-terminal domain of E2, responsible for the activation of transcription and replication and genome segregation, is replaced by the short E8 domain [15]. The E8^E2 protein is a repressor of the initial genome amplification and viral gene expression in undifferentiated cells [15]. In addition to DNA binding, E8^E2’s repressive activities require the E8 domain which mediates the interaction with cellular, multi-protein NCoR/SMRT co-repressor complexes composed of NCoR and/or its homologue SMRT, TBL1 and/or its homologue TBLR1, HDAC3 and GPS2 proteins [16–18]. NCoR/SMRT complexes are recruited by a variety of cellular transcription factors to mainly inhibit gene expression and HDAC3 is regarded as the main repression activity of the complex as it deacetylates lysine residues in histones and non-histone proteins [19,20]. Interestingly, repression by E8^E2 is NCoR/SMRT-dependent, but largely HDAC3-independent in reporter assays [16,18,21]. The inhibition of genome replication by E8^E2 has been suggested to be part of a copy number control mechanism, but it was unclear why the genome copy number needs to be restricted in undifferentiated keratinocytes. A recent study revealed that the Mus musculus PV1 (MmuPV1) E8^E2 protein acts as an NCoR/SMRT-dependent repressor of viral gene expression and replication comparable to its HPV counterparts [17]. Surprisingly, MmuPV1 E8^E2 knock-out genomes or genomes encoding an NCoR/SMRT-binding deficient E8^E2 protein do not induce tumors in immune-deficient nude mice in contrast to wild-type MmuPV1 [17,22]. This suggested that increased genome replication and viral protein expression in basal keratinocytes per se prevents tumor formation. Interestingly, a large fraction of keratinocytes transfected with E8^E2 mt genomes, expressed the viral late E4 protein in monolayer culture suggesting that these cells have entered the productive replication cycle. E4-expressing cells displayed a cell cycle shift consistent with findings that HPV E4 proteins can induce a G2-arrest in proliferating cells [17,23]. Taken together, these findings suggested that the loss of E8^E2 activity inappropriately forces cells into the productive replication cycle in undifferentiated keratinocytes which prevents infected cells from proliferating and expanding in the basal layer and therefore tumor formation in vivo.

Remarkably, HPV differ in their ability to establish E8^E2 mt genomes as episomes in stable keratinocyte cell lines. Whereas HPV16 and beta-HPV 49 E8^E2 mt genomes can be stably maintained as high copy number episomes, HPV31 E8^E2 mt genomes often fail to induce proliferating keratinocyte colonies and the rare colonies, that give rise to stable cell lines, display exclusively integrated HPV31 genomes [24–27].

We now demonstrate that HPV31 E8^E2 mt genomes induce genome amplification and E4 expression in undifferentiated keratinocytes which prevents infected cells from proliferating and therefore colony formation. This also explains the outgrowth of cell lines with integrated viral E8^E2 mt genomes as late gene expression cannot be induced from integrated HPV genomes [28]. Interestingly, the knock-down of NCoR/SMRT complex components NCoR/SMRT and TBL1/TBLR1, but not HDAC3, in undifferentiated keratinocytes stably maintaining HPV31 episomes mimics the loss of E8^E2 and induces genome amplification and E4 expression. In contrast, only minor differences in the levels of E4 expression and late gene transcription can be observed in differentiated cells. This strongly suggests that E8^E2 has evolved to prevent genome amplification and E4 expression in undifferentiated cells and that a differentiation-dependent inactivation of E8^E2 contributes to productive replication. These findings also highlight a potential antiviral strategy that could eliminate infected cells by forcing them into the productive replication cycle.

## Results

### HPV31 E8^E2 mt genomes induce late gene transcription and E4 protein expression in transiently-transfected human keratinocytes

**Fig 1:**
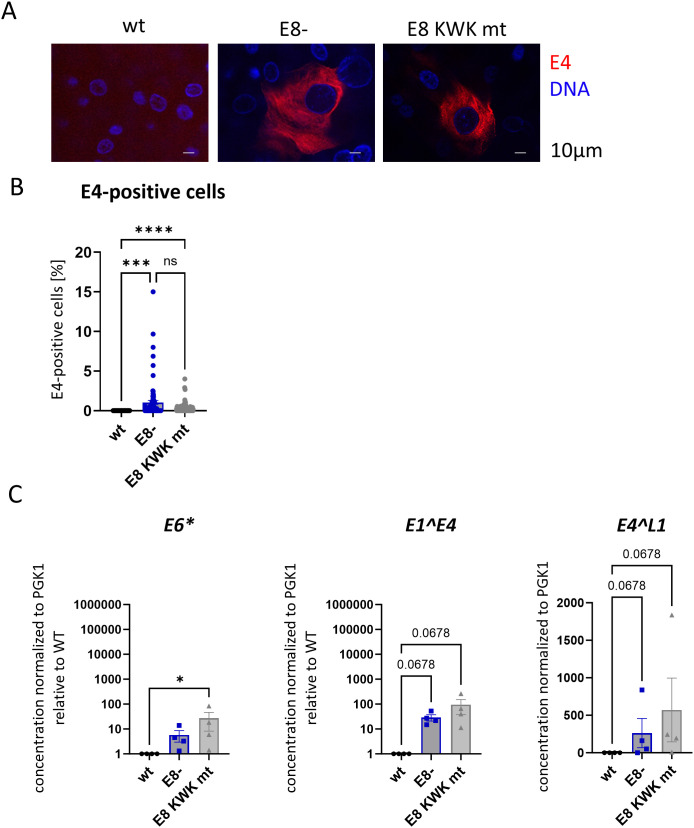
(A) Representative images of NHK transfected with HPV31 wt, E8-, or E8 KWK mt genomes and analyzed for HPV31 E4 protein expression 6d p.t. by IF staining. Nuclei were stained with DAPI. The scale bars are 10 µm. **(B)** Quantification of the fraction of E4-positive cells using random images at a 200x magnification. Please note that no E4-positive cells were detected in wt-transfectants. Statistical significance was determined by a mixed-effects analysis with Tukey’s multiple comparisons test (n = 50 pictures in 6 different donors for the wt, n = 80 pictures each in 9 different donors for E8- and E8 KWK mt; *** p = 0.001; **** p < 0.0001). Error bars indicate the SEM. **(C)** QPCR analysis of spliced viral transcripts E6*, *E1^E4*, and *E4^L1*. Values were normalized to PGK1, set relative to the wild-type (with the exception of *E4^L1* as no signal was obtained for the wt) and analyzed by a Friedman test with Dunn’s multiple comparisons test (*p = 0.05) or one-way ANOVA with Tukey’s multiple comparisons test. Error bars indicate the SEM.

Previous studies have shown that HPV31 E8- (a start codon mutation preventing E8^E2 expression, originally labelled E8 ATG) and E8 KWK mt (encoding an NCoR/SMRT complex-binding deficient E8^E2 mutant (mt)) genomes replicate to much higher levels in transient replication assays [24,27,29]. To test if HPV31 E8- and E8 KWK mt induce E4 expression and late gene transcription, re-circularized wild-type (wt) and mt genomes were transiently transfected into normal human keratinocytes (NHK). To detect cells entering the productive replication cycle, we performed immunofluorescence (IF) analysis 6 d post transfection with the E4 monoclonal antibody FH1.1 [30]. Consistent with our prediction, E4-expressing cells could be detected when E8- or E8 KWK mt genomes were transfected, but not with the wild-type (Fig 1A). The numbers of E4-positive cells were quantified from random images which revealed that both E8- and E8 KWK mt genomes displayed significantly higher numbers of E4-expressing cells than the wt (Fig 1B). To further confirm that the inactivation of E8^E2 not only induces E4 protein expression, but also late gene transcription, splice junctions, which are part of early and late viral transcripts were quantified by qPCR analysis (Fig 1C). E6* containing transcripts, derived from the major early P97 promoter, were present at 5.7-fold and 27.3-fold increased levels in E8- and E8 KWK mt transfected cells compared to the wt (Fig 1C). Remarkably, *E1^E4* containing transcripts, which can be derived from P97 or PL, were induced by HPV31 E8- and E8 KWK mt genomes 29.1-fold and 94.9-fold, respectively. This is consistent with the previous RNAse protection analyses demonstrating that PL is induced upon inactivation of E8^E2 [29]. *E4^L1* containing transcripts, which are mainly derived from PL, could only be detected in E8- and E8 KWK mt transfected and therefore a relative induction compared to the wt could not be calculated (Fig 1C). Taken together, these results indicate that E8^E2 mt genomes induce E4 protein expression and PL transcripts to a greater extent than P97 transcripts consistent with an entry into the productive phase.

### The E4 and E5 genes are not responsible for the inability of HPV31 E8^E2 knock-out genomes to become established as episomes in stable keratinocyte cell lines

**Fig 2:**
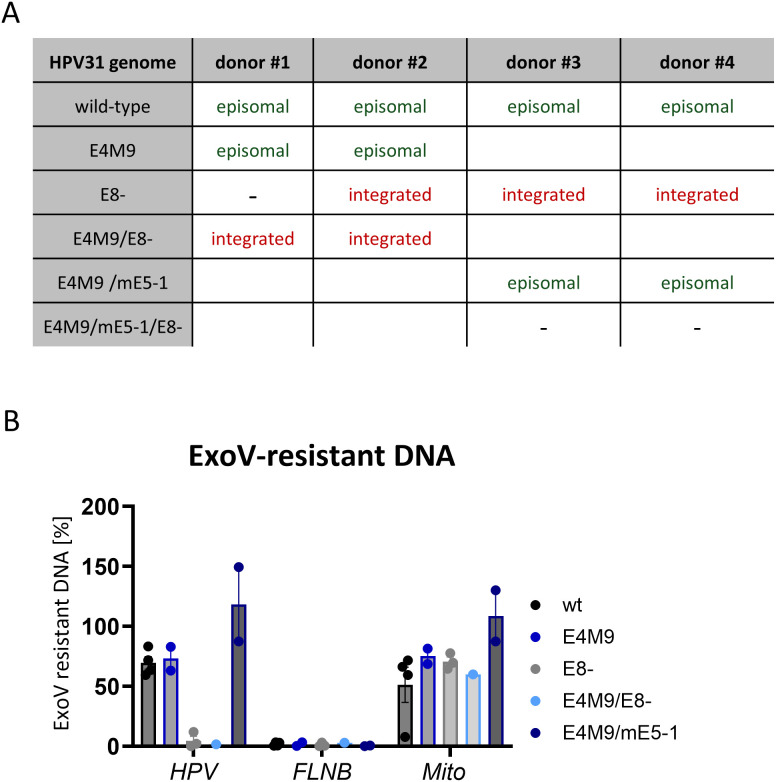
(A) Tabular results of immortalization assays and the physical state of viral genomes (integrated/episomal) in stable cell lines as determined in (B) using different HPV31 genomes and different donors as indicated. (–) indicates that no colonies could be expanded into a cell line. **(B)** QPCR analysis of exonucleaseV-digested, total DNA isolated from the different HPV31 positive cell lines. Fractions of exonucleaseV-resistant HPV31 using an amplicon in *E2*, the cellular filaminB gene (*FLNB)* and mitochondrial DNA (Mito) to account for the linearization of DNA during the isolation process were determined.

Our previous analyses suggested that the failure of MmuPV1 E8^E2 mt infected cells to proliferate could be due to an E4 protein-mediated G2 arrest [17]. To test if the expression of the HPV31 E4 protein prevents the establishment of E8- genomes as episomes in NHK, we introduced the previously described HPV31 E4M9 mutation into the E8- and wt background [31]. E4M9 introduces a translation termination at codon 10 of E4 to disrupt E4 protein expression, but is silent in the overlapping E2 gene [31]. NHK from two donors were transfected with re-circularized wt, E4M9, E8-, or E8-/E4M9 genomes and a drug-selectable plasmid. Wt, E4M9, and E8-/E4M9 genomes gave rise to drug-resistant colonies from both donors that were pooled each and expanded as stable cell lines (Fig 2A). A stable cell line with E8- genomes could be established in this experiment only from donor #2 (Fig 2A). The physical state of viral genomes in stable cell lines was analyzed by qPCR after exonucleaseV digest to remove integrated viral genomes [32]. Mitochondrial DNA, which is exclusively extrachromosomal, was used to determine the extent of linear DNA arising during the purification process and the cellular filamin B (*FLNB*) gene to determine the efficiency of the exonucleaseV digest. This analysis revealed that wt and E4M9 genomes are present at exonucleaseV-resistant DNA levels similar to mitochondrial DNA, in contrast to the cellular *FLNB* gene, indicating episomal maintenance (Fig 2B). This confirms previous observations that the inability to express E4 does not impact HPV31 maintenance replication in undifferentiated keratinocytes [31]. In contrast, E8- and E8-/E4M9 cell lines revealed very low levels of exonucleaseV-resistant viral genomes indicating integrated viral genomes. This suggested that E4 protein expression is not responsible for the failure of E8- genomes to become established as episomes. The major PL *E1^E4, E5* transcript encodes, in addition to E4, the E5 protein. To investigate a contribution of E5 to the E8- phenotype, the previously characterized E5 mutation mE5-1, which inactivates the E5 start codon to disrupt E5 expression [33], was introduced in the E4M9 or E8-/E4M9 background. NHK from two donors were transfected with re-circularized viral genomes as described above. Only wt, E4M9/mE5-1, and E8- genomes gave rise to cell lines (Fig 2A). As expected, wt and E8- genomes were present as episomes and integrates, respectively. E4M9/mE5-1 genomes were also present as episomes (Fig 2B), confirming that neither the E4 nor the E5 knock-out impacts episomal maintenance of HPV31 [31,33]. No colonies could be expanded from E8-/ E4M9/mE5-1 transfectants suggesting that the failure of E8^E2 mt genomes to become established as episomes is not dependent on E4 and E5 protein expression.

### Depletion of NCoR/SMRT complex components in HPV31-positive cells induces genome amplification and E4 expression

While these studies provide evidence that E8^E2 is important to limit the initial amplification phase after transfection of genomes in order to prevent E4 protein and late gene expression, it is not known, if E8^E2 prevents entering the productive replication cycle in undifferentiated cells undergoing maintenance replication. To address this, we depleted NCoR/SMRT complex components in cell lines maintaining episomal HPV31 genomes: HK-HPV31, established by transfection of NHK with HPV31 wt genomes, and the CIN612-9E cell line established from a HPV31-positive cervical intraepithelial 1 lesion [27,34]. While the functionally redundant TBL1 and TBLR1 proteins, core components of NCoR/SMRT complexes, have been both identified as interactors of E8^E2 proteins [16–18], their individual expression patterns and contributions to E8^E2 repression in cell lines maintaining HPV episomes have not been addressed. We therefore first confirmed that TBL1 and TBLR1 transcripts are expressed in HK-HPV31 and CIN612-9E cell lines using gene-specific primers validated with TBL1 and TBLR1 expression constructs. TBL1 and TBLR1 expression could be efficiently reduced by transfecting siRNA pools targeting TBL1 or TBLR1 (S1A Fig). Immunoblot analyses with two different monoclonal antibodies showed distinct detection patterns. The Santa Cruz antibody #sc137006 recognized both flag-tagged TBL1 and TBLR1 upon overexpression, whereas the Cell Signaling antibody #74499 detected only TBLR1 (S1B Fig). Notably, both antibodies detected a prominent ~56 kDa band in control-transfected cells, consistent with the predicted size of endogenous TBLR1 (55.6 kDa). In CIN612-9E cells, immunoblotting revealed a dominant 56 kDa species, with the cross-reactive antibody also detecting minor higher-molecular-weight forms (S1C Fig). SiRNA knockdown experiments further clarified protein identity. Individual siRNA pools specifically reduced their respective transcripts (S1A Fig). Knockdown of TBLR1 efficiently depleted the 56 kDa band detected by both antibodies, indicating that this species corresponds primarily to TBLR1. However, siTBL1 also reduced this band when detected with the cross-reactive antibody, suggesting a minor contribution from TBL1 (S1C Fig). Together, these results indicate that TBLR1 is the predominant form expressed in HPV31-positive cells, while TBL1 is present at lower levels. The presence of a ~ 56 kDa TBL1 species is consistent with alternatively spliced transcripts encoding an N-terminally truncated isoform of approximately 526 amino acids (predicted molecular weight ~57 kDa; ENST00000424279.6, ENST00000380961.5, ENST00000647060.1).

Only the combined knock-down of TBL1 and TBLR1 significantly induced *E1^E4* transcripts, consistent with both contributing to the repression activity of E8^E2 (S1D Fig). In addition, previously validated siRNA pools against HDAC3 or NCoR and SMRT were transfected and knock-down efficiency determined by qPCR with gene specific primer pairs (S2 Fig). All targeted components could be reduced in both cell lines, but the knock-down was generally more efficient in CIN612-9E cells.

**Fig 3:**
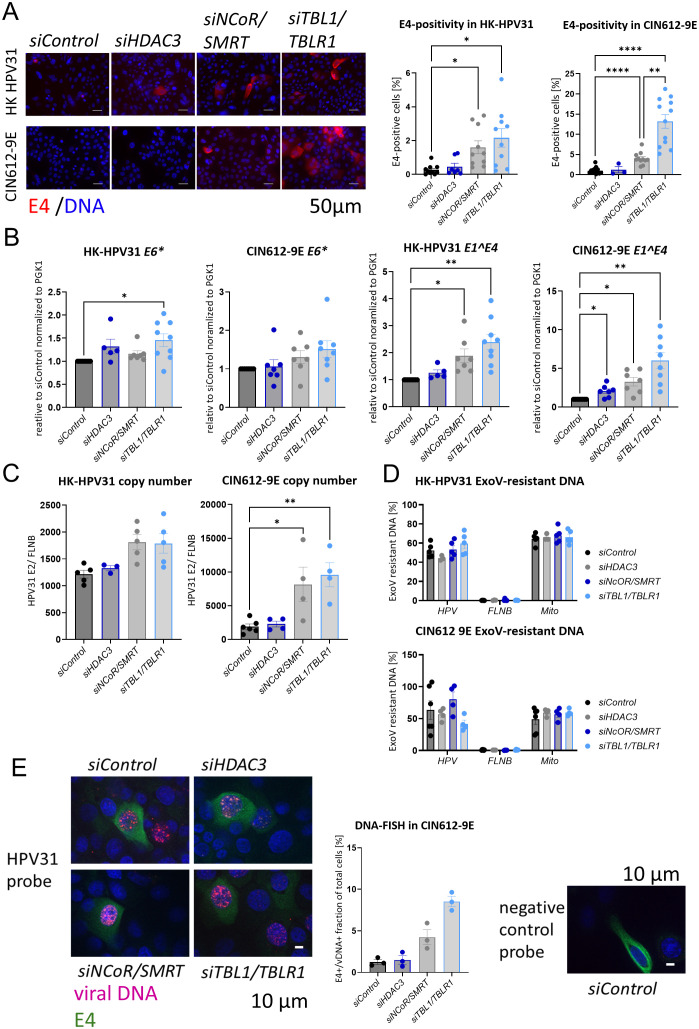
Analysis of HK-HPV31 and CIN612-9E transfected for 48 h with different siRNAs as indicated. **(A)** Representative IF images of HPV31 E4. Nuclei were stained with DAPI. Magnification is 200x, the scale bars are 50 µm. The graphs on the right represent the quantification of E4-expressing cells in each cell line using random images. Statistical analysis was done by Mixed effects analysis with Tukey’s multiple comparisons test (n = 7-10 for HK-HPV31 wt, n = 3-13 for CIN612-9E; *p = 0.05; *** p = 0.001; **** p < 0.0001). Error bars indicate the SEM. **(B)** QPCR analysis of spliced viral transcripts E6* and *E1^E4*. Values were normalized to *PGK1* and are expressed relative to siControl. Data was analyzed using the mixed effects analysis with the Dunnett’s multiple comparisons test (n = 5-9; *p < 0.05; **p < 0.01). Error bars indicate the SEM. **(C)** QPCR analysis of viral genome copies relative to the cellular *FLNB* gene. Statistical significance was analyzed by ordinary one-way ANOVA with Tukey’s multiple comparisons test (n = 3-6; *p = 0.05; **p = 0.01). Error bars indicate the SEM. **(D)** QPCR analysis of exonucleaseV-digested total DNA. The fraction of exonucleaseV-resistant DNA was determined for HPV31 using an amplicon in E2, the cellular filaminB gene (FLNB) and mitochondrial DNA (Mito) to account for the linearization of DNA during the isolation process. **(E)** DNA-FISH/E4-IF images of CIN612-9E cells, 48h post transfection with different siRNAs as indicated. Representative IF images of viral DNA and HPV31 E4-positive cells were captured with 630x magnification (Scale bar = 10 µm). DNA was stained DAPI. The graph in the middle shows the quantification of the fraction of DNA-FISH/E4-positive cells from three independent experiments. To confirm specificity of the HPV31 probe, a negative control probe was included and is shown on the right.

Both cell lines were seeded in the presence of mitomycin C-treated NIH 3T3 J2 fibroblasts at low density, transfected with the different siRNAs, and the fraction of E4-positive cells was determined by quantitative IF of random images 48h post transfection. E4-positivity in siControl transfected cells was 0.26% and 1.0% in HK-HPV31 and CIN612-9E, respectively (Fig 3A). Depletion of HDAC3 resulted in 0.46% and 1.23% E4-positivity in HK-HPV31 and CIN612-9E, respectively, but these minor increases were not statistically significant (Fig 3A). In contrast, depletion of NCoR and SMRT significantly increased the fraction of E4-positive cells to 1.1% (HK-HPV31) and 4% (CIN612-9E) and the depletion of TBL1 and TBLR1 to 2.2% (HK-HPV31) and 13.2% (CIN612-9E) (Fig 3A). QPCR analysis of spliced viral transcripts reveled only minor changes of E6* upon knock-down of NCoR/SMRT complex components, whereas *E1^E4* was significantly induced by NCoR/SMRT or TBL1/TBLR1 knock-down in both cell lines (Fig 3B). Knock-down of HDAC3 also significantly induced *E1^E4* in CIN612-9E, but to lower levels than NCoR/SMRT or TBL1/TBLR1 knock-downs, consistent with a minor contribution of HDAC3 to repression. Consistent with a more efficient knock-down (S2 Fig), induction levels were generally higher in CIN612-9E than in HK-HPV31 cells. QPCR analysis of viral genome copy numbers determined by the ratio of *E2* copies to cellular *FLNB* gene copies indicated a significant increase by NCoR/SMRT and TBL1/TBLR1 knock-down in both cell lines (Fig 3C). ExonucleaseV-resistance assays revealed that viral genomes were resistant at similar levels as mitochondrial DNA in HK-HPV31 and in CIN612-9E cells suggesting mainly extrachromosomal viral genomes (Fig 3D). The knock-down of HDAC3 resulted in slightly increased integration levels in HK-HPV31, whereas the knock-down of TBL1/TBLR1 and of HDAC3 slightly increased integration levels in CIN612-9E (Fig 3D). Taken together, these data do not suggest that the activation of genome amplification by NCoR/SMRT complex depletion results consistently in enhanced integration of viral genomes. The combination of E4 immunofluorescence with HPV31 DNA-fluorescence in situ hybridization revealed multiple nuclear dots in E4-positive cells in siControl-, siHDAC3-, siNCoR/SMRT-, and siTBL1/TBLR1-transfected cells consistent with the phenotype of viral replication factories in which vegetative genome amplification occurs (Fig 3E) [35–37]. A quantification revealed that depletion of NCoR/SMRT or TBL1/TBLR1 elevated the number of DNA-FISH/E4-double positive cells (Fig 3E) suggesting that the increase in viral copy numbers measured by qPCR is mainly a consequence of cells undergoing vegetative genome amplification. Taken together, these data strongly suggest that the depletion of NCoR/SMRT complex components from HPV31-positive cells induces genome amplification and E4 expression in cells maintained as a monolayer.

### E4 expression is induced in basal-like keratinocytes

**Fig 4:**
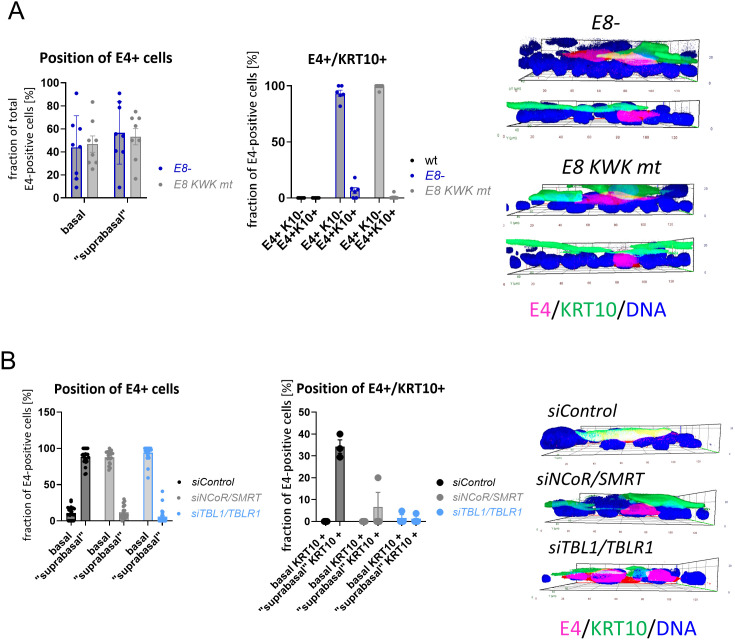
(A) 3D image analysis of NHK transiently transfected with HPV31 wt, E8- and E8 KWK mt genomes stained for E4 and KRT10. On the right, representative 3D images of E8- and E8 KWK mt genome transfected cells showing E4-positive cells attached to the plate or growing on top. On the left, quantification of the fraction of E4-positive cells attached to the culture plate (designated as “basal”) or growing on top (designated as “suprabasal”) (n = 6-8). In the middle, quantification of E4-positive cells expressing KRT10 (n = 6). **(B)** 3D image analysis of the fraction of E4-positive cells in CIN612-9E cells 48h after siRNA-transfection. Representative images are shown on the right and the bar graph on the left shows the quantification of E4-positive cells and their relative position. In the middle, quantification of KRT10-expression in E4-positive cells is shown.

It is generally accepted that productive HPV replication requires keratinocyte differentiation. The observation that genome amplification and E4 expression can be induced by transfection of HPV31 E8^E2 mutant genomes in NHK, or by depletion of NCoR/SMRT complex components in stable cell lines maintained as undifferentiated monolayers, raised the question of whether E8^E2 inactivation bypasses the need for differentiation. NHK were transfected with re-circularized wt, E8-, or E8 KWK mt genomes and stained 6d p.t. for E4 and the suprabasal keratinocyte differentiation marker keratin 10 (KRT10). Three-dimensional reconstruction of microscopic images was used to determine whether E4-expressing cells remained attached to the culture plate (basal phenotype) or grew on top of other cells (suprabasal phenotype). Consistent with KRT10 being a suprabasal differentiation marker, only cells growing on top of others were KRT10-positive. Remarkably, 44% and 47% of E4-expressing keratinocytes in E8- and E8 KWK mt transfected cultures were attached to the culture plate and more than 93% of those were KRT10-negative (Fig 4A). These results indicate that HPV31 E8^E2 mt can enter the productive replication cycle in basal keratinocytes, independently of differentiation. As pointed out above, no E4-expressing cells were observed in wt-transfected cells and so their status could not be analyzed.

**Fig 5:**
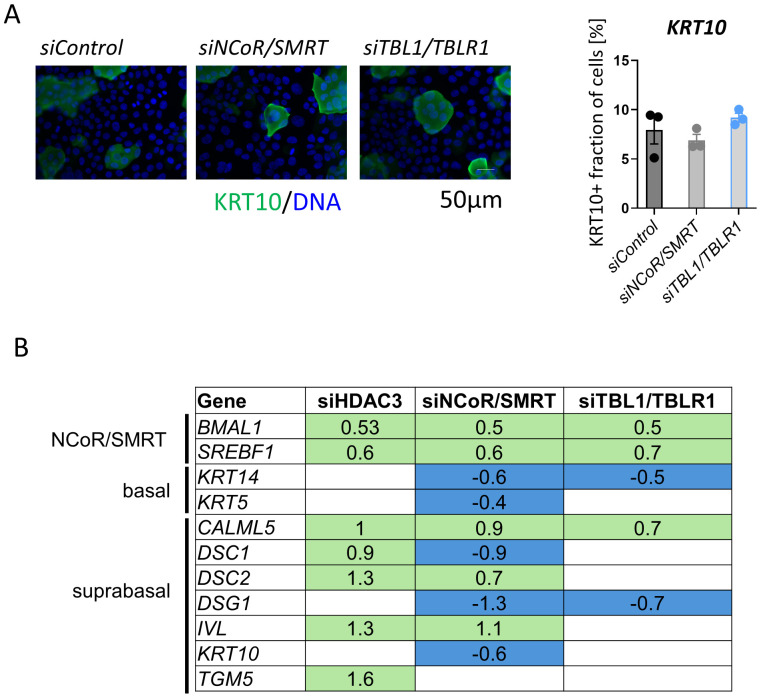
Analysis of keratinocyte differentiation markers in siRNA-transfected CIN612-9E cells. **(A)** IF analysis of KRT10 expression with representative images shown on the left. Magnification is 200x, the scale bar is 50µm and DNA was stained with DAPI. The respective quantification is on the right. **(B)** Differential gene expression analysis of selected genes in an RNA-seq data set of siRNA-transfected CIN612-9E (n = 3). Values represent log2-fold changes compared to the siControl with an adjusted p-value <0.1.

Analysis of E4-expressing cells in siControl-transfected HK-HPV31 or CIN612-9E cells confirmed that only a minority remained attached to the plate. Over 90% occupied a suprabasal position, and a significant fraction expressed KRT10, consistent with previous findings that HPV31 E4 expression from wt genomes occurs predominantly in differentiated keratinocytes (Figs 4B, S3). In sharp contrast, in cells transfected with siNCoR/SMRT or siTBL1/TBLR1, more than 87% of E4-expressing cells remained attached to the culture plate and did not express KRT10 (Figs 4B, S3). A small fraction of suprabasal E4-positive cells induced by E8^E2 inactivation also expresses KRT10 (Fig 4A, B), indicating that these cells retain the capacity for differentiation-dependent KRT10 expression. IF analysis of CIN612-9E cells indicated that KRT10 expression was not noticeably altered by siNCoR/SMRT or siTBL1/TBLR1 transfection (Fig 5A) suggesting that depletion of NCoR/SMRT complexes does not strongly impact keratinocyte differentiation. To further investigate this, we analysed the differential expression of specific transcripts linked to keratinocyte differentiation or regulated by NCoR/SMRT complexes in an RNA-seq data set of siHDAC3-, siNCoR/SMRT-, or siTBL1/TBLR1-transfected CIN612-9E cells. This revealed that the NCoR/SMRT-complex target genes *SREBP1* [38] and *BMAL1* [39,40] are induced by depletion of individual components to similar levels confirming the effects of NCoR/SMRT complexes on known targets (Fig 5B). In contrast, the analysis of basal (*KRT5, KRT14*) and suprabasal keratinocyte differentiation genes (*KRT1, KRT10, DSC1, DSC2, DSG1, FLG, IVL, LCE1A, TGM5, PRR9, CALML5, CASP14, MAF, MAFB)* [41] revealed no significant changes compared to the control (padj<0.1) of *CASP14, FLG, KRT1, LCE1A, MAF, MAFB,* and *PRR9*. SiHDAC3 induced the suprabasal markers *CALML5, DSC1, DSC2, IVL,* and *TGM5* (Fig 5B). The basal keratinocyte marker *KRT14* and the suprabasal marker *DSG1*, were reduced by both siNCoR/SMRT and siTBL1/TBLR1, whereas the suprabasal marker *CALML5* was induced (Fig 5B). *KRT10* and *DSC1* were reduced and *DSC2* and *IVL* were induced by siNCoR/SMRT but not regulated by siTBL1/TBLR1 (Fig 5B).

Taken together, the lack of a consistent induction of suprabasal differentiation markers by siNCOR/SMRT or siTBL1/TBLR1 suggests that genome amplification and E4 expression are not driven by activation of keratinocyte differentiation. These results indicate that inactivation of E8^E2, either by mutation or depletion of NCoR/SMRT complexes, promotes entry into the productive replication cycle in basal-like keratinocytes, effectively phenocopying differentiation.

### Cells with induced E4 expression are preferentially, but not arrested, in the G2 phase of the cell cycle

**Fig 6:**
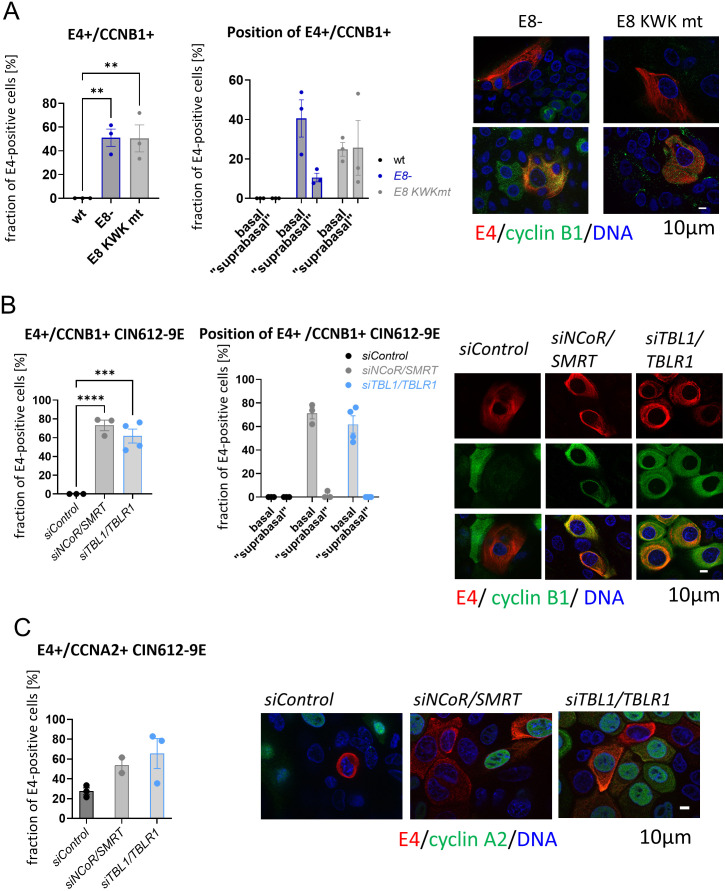
IF analysis of cyclin B1 (CCNB1) and cyclin A2 (CCNA2) expression in E4-positive cells 48h after siRNA transfection. **(A)** Representative images of E4 + /CCNB1- and E4 + /CCNB1 + NHK transiently transfected with E8*-* and E8 KWK mt genomes 6 d p.t.. Magnification is 630x, the scale bar is 10µm and DNA was stained with DAPI. On the left, the fractions of E4 + /CCNB1 + cells are depicted. Statistical analysis was done by ordinary one-way ANOVA with Dunnett’s multiple comparisons test (n = 3, **p = 0.01). In the middle, quantification of these cells as fraction of E4-positive cells attached to the culture plate (designated as “basal”) or growing on top (designated as “suprabasal”). **(B)** Analysis and representative images of CIN612-9E cells 48h after siRNA-transfection. On the right, representative images of E4 + /CCNB1 + cells are depicted. Magnification was 630x, the scale bar is 10µm and DNA was stained with DAPI. On the left, quantification of the IF staining is shown. Statistical analysis was done by ordinary one-way ANOVA with Dunnett’s multiple comparisons test (n = 3-4, *** p = 0.001, **** p < 0.0001). In the middle, the respective fraction of E4-positive cells attached to the culture plate (designated as “basal”) or growing on top (designated as “suprabasal”) is shown. **(C)** IF analysis of CIN612-9E stained for HPV31 E4 and cyclin A2 (CCNA2). The quantification of E4-positive cells, that also express CCNA2, is shown in a graph on the left. Representative images of cells stained for E4, CCNA2, and DNA are shown on the right. Magnification is 630x, the scale bar is 10µm and DNA was stained with DAPI.

**Fig 7:**
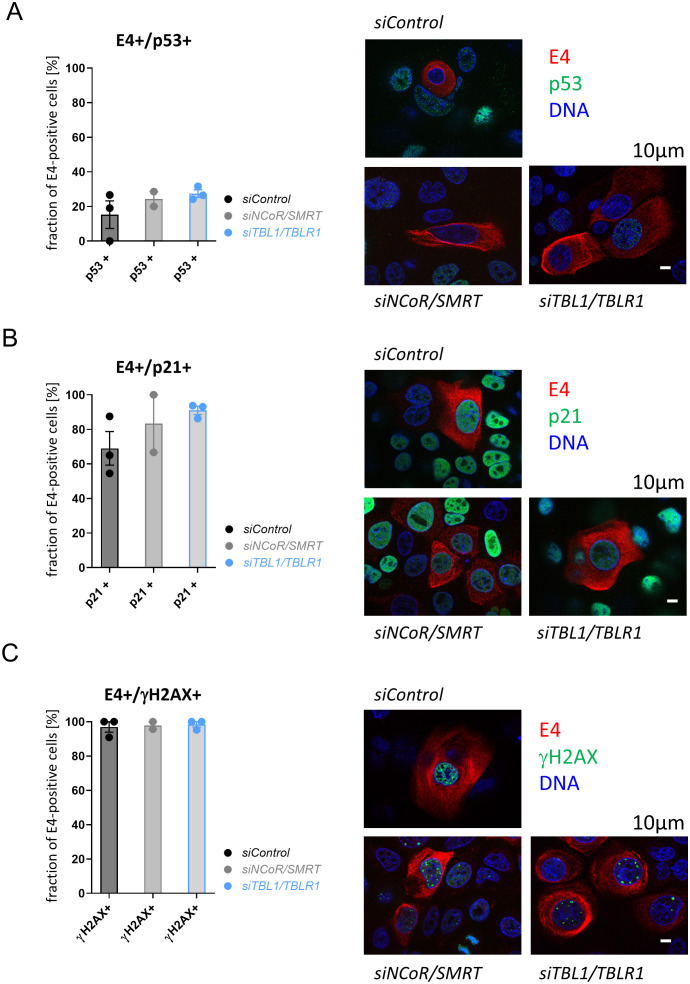
IF staining of CIN612-9E 48 h after siRNA transfection. HPV31 E4 was stained in combination with p53 **(A)**, p21 **(B)**, or γH2AX **(C)**. Graphs on the left are quantifications of the fraction of E4 + /marker+ cells. Representative IF images are shown on the right. DNA was stained with DAPI, 630x magnification, scale bar 10µm.

Previous studies suggested that HPV18 E4-expressing cells are in the G2-phase of the cell cycle, but eventually exit the cell cycle [42,43]. Transfection of E8- or E8 KWK mt genomes resulted in 51% of E4-positive cells with cytoplasmic cyclin B1 expression indicative for the G2 phase (Fig 6A). Upon knock-down of NCoR/SMRT or TBL1/TBLR1 in HK-HPV31 or CIN612-9E cells, the majority of E4-expressing cells also showed cytoplasmic cyclin B1 expression (Figs 6B, S4). Only very few E4-expressing cells in siControl-transfected HK-HPV31 or CIN612-9E cells showed cytoplasmic cyclin B1 expression suggesting differences between the productive replication cycle physiologically induced or induced by E8^E2 inactivation. To further analyze this, CIN612-9E cells were co-stained for E4 and cyclin A2, which is expressed from the S-phase until the G2-phase [44]. This revealed that 27.3% of E4-positive cells in the siControl, 53.7% in NCoR/SMRT-, and 65.5% in TBL1/TBLR1-depleted cells express cyclin A2 (Fig 6C). This confirms that a larger fraction of E4-positive cells is in S and G2 in NCoR/SMRT- or TBL1/TBLR1-depleted cells. To exclude that the knock-down of TBL1 and TBLR1 changes the expression of cyclin A2 and cyclin B1 through other mechanisms, keratinocytes expressing HPV31 E6/E7 from the pLXSN retroviral vector were transfected with siControl or siTBL1/TBLR1 and cyclin A2 and cyclin B1 protein levels were determined by immunoblot. This revealed that cyclin A2 and cyclin B1 levels were unchanged, despite a significant knock-down of TBLR1 protein levels (S5 Fig) suggesting that the increased expression of G2 phase markers in E4-positive cells is specific for cells with replicating viral genomes. In summary, these data suggested that cells induced to enter the productive replication cycle might be arrested in the G2-phase. We therefore investigated cell cycle regulators in CIN612-9E cells whose expression is modulated by HPV such as p53, which is degraded by E6, p21/CDKN1A, whose expression is inhibited by E6 via p53 and activated by E7, and phospho-S139-H2AX (γH2AX), a marker for DNA damage, which is induced by high risk HPV [10,45,46]. This revealed that only a minority of E4-positive cells expresses p53 (15.2%), whereas the majority is p21-positive (69.0%) and almost all E4-expressing cells are γH2AX-positive (97.0%) (Fig 7). This suggests that E6 and E7 are active in E4-expressing cells. Nevertheless, no major differences between the control and depletion of NCoR/SMRT or TBL1/TBLR1 were observed in E4-expressing cells indicating that the expression of p53, p21, and γH2AX is not linked to the increased expression of cyclin A2 and B1.

**Fig 8:**
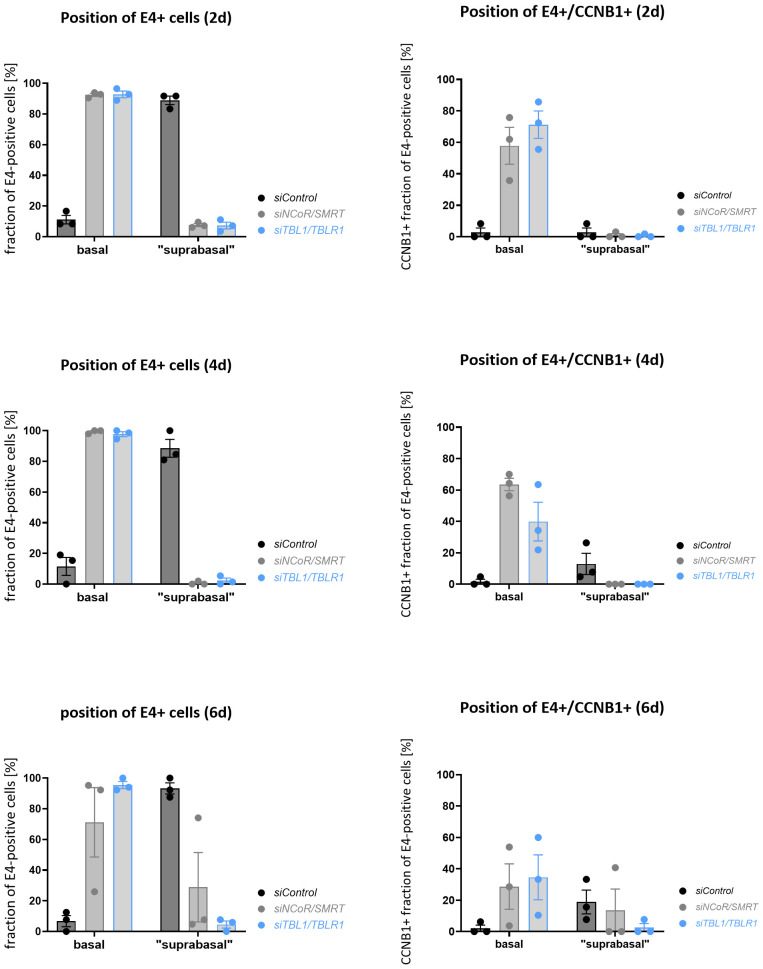
IF analysis of CIN612-9E 2 d, 4 d and 6 d after siRNA transfection. On the left, the fractions of E4-expressing cells and their relative position to the cell culture surface (basal/ “suprabasal”) 2/4/6 d after siRNA-treatment are depicted. On the right, the fractions of E4-positive cells expressing CCNB1 and their relative position to the cell culture surface (basal/ “suprabasal”) are shown.

These experiments were carried out 2d post siRNA transfection. To investigate the fate of E4-expressing cells over time, siRNA-transfected CIN612-9E cells were analyzed 2d, 4d, and 6d post transfection. QPCR analyses indicated that the respective transcript levels remained low after siRNA transfection during the observation period (S6 Fig). Interestingly, a large fraction of E4-positive cells remained basal in NCoR/SMRT- or TBL1/TBLR1-depleted cells, but the fraction of E4/cytoplasmic cyclin B1-double positive cells decreased over time (Fig 8). This suggest that these cells are not permanently arrested in G2, but are able to exit the cell cycle comparable to the control.

### Depletion of NCoR/SMRT complexes in differentiated cells does not further enhance HPV31 late gene and E4 protein expression

**Fig 9:**
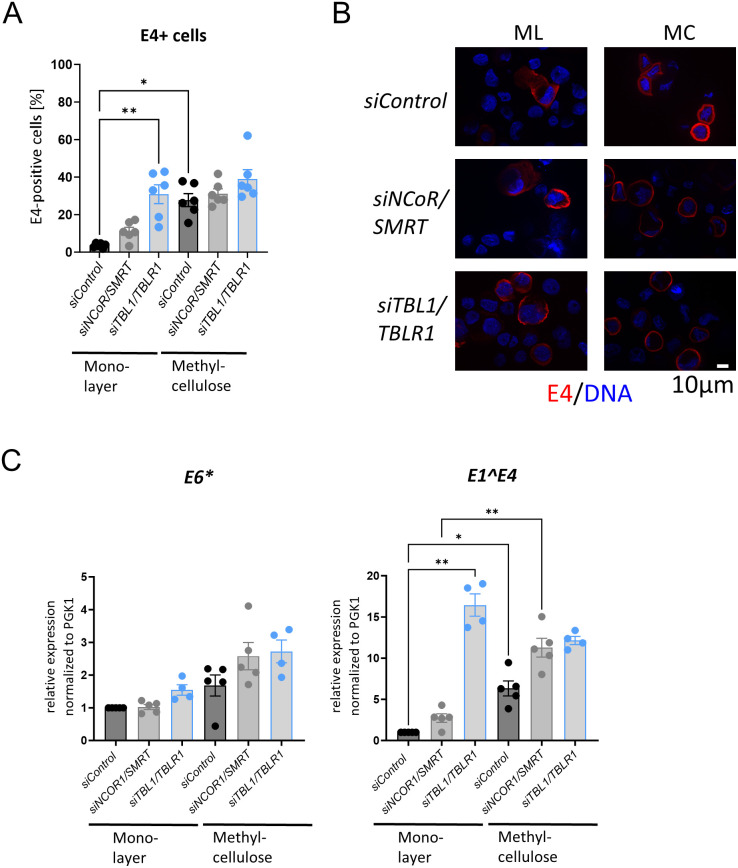
Comparison of siRNA-transfected CIN612-9E cells grown in monolayer (ML) or differentiated in methyl cellulose medium (MC) for 48 h. **(A)** Fraction of E4-positive cells determined by IF staining in monolayer culture and after differentiation in methylcellulose medium. Statistical analysis for the E4-expression was done by Kruskal-Wallis test with Dunn’s multiple comparisons test (*p = 0.05, **p = 0.01). **(B)** Representative images of E4-IF staining. Magnification is 630x, scale bar 10µm and DNA was stained with DAPI. **(C)** QPCR analysis of viral spliced transcripts E6* and *E1^E4.* Values were normalized to *PGK1* and set relative to the ML siControl. Statistical analysis was done by a mixed-effects analysis with the Tukey’s multiple comparisons test (n = 4-5 experiments). **(A)**, **(C)** Error bars indicate the SEM.

The results so far indicated that depletion of NCoR/SMRT complexes can overcome the requirement for differentiation to induce the productive replication cycle, therefore the question remained if differentiation targets E8^E2 activity. To investigate this, siRNA-transfected CIN612-9E cells were differentiated for 48h by suspension in methyl cellulose medium. QPCR analysis indicated that the siRNA knock-down efficiency is similar in monolayer and methyl cellulose-differentiated cells (S7 Fig). Quantitative IF analysis revealed an increase of E4-positive cells in siControl-transfected cells upon differentiation, as expected (Fig 9A, B). Remarkably, while siTBL1/TBLR1 greatly enhanced the fraction of E4-positive cells compared to the control in undifferentiated cells, only a weak additional increase was observed in differentiated cells. Consistent with this, the fraction of E4-positive siNCoR/SMRT-transfected cells was comparable to the control upon differentiation (Fig 9A, B). The analysis of viral gene expression by qPCR revealed that E6* levels were slightly increased by differentiation in siControl-, NCoR/SMRT, and TBL1/TBLR1-transfected cells (Fig 9C). Consistent with increased E4 protein expression, *E1^E4* levels were increased in siControl-transfected cells by differentiation (Fig 9C). Differentiation further increased *E1^E4* levels in NCoR/SMRT-depleted cells to slightly higher levels than the control. While TBL1/TBLR1-depletion already increased *E1^E4* levels in undifferentiated cells, there was no further increase upon differentiation, but rather a slight decrease to levels similar in NCoR/SMRT depleted cells and slightly higher than in the control. Taken together, these data indicate that, in contrast to undifferentiated cells, differentiation attenuates the effects of NCoR/SMRT complex knock-down on E4 protein expression and late gene transcription. This suggests that E8^E2 activity is most likely partially inactivated by differentiation to allow productive replication.

## Discussion

Productive replication of HPV takes only place in suprabasal keratinocytes that have entered their terminal differentiation program [2–4]. It is characterized by the formation of viral replication centers in which vegetative genome replication occurs and the activation of the differentiation-dependent viral PL which drives the expression of the abundant *E1^E4, E5* transcript resulting in E4 protein expression. Cells which have entered productive replication are predominantly in the G2 phase and will eventually exit from the cell cycle, but do not undergo mitosis and cell division.

PV express the conserved E8^E2 repressor protein and E8^E2 mt genomes have been shown to display increased genome replication in monolayer culture short-term assays indicating that E8^E2 controls viral copy number in the absence of keratinocyte differentiation, but the reason for limiting copy number has not been clear. Surprisingly, MmuPV1 E8^E2 mt genomes do not form tumors in vivo and this has been linked to the expression of the E4 protein in cultured, undifferentiated keratinocytes which is able to induce an G2 phase arrest in proliferating cells [17,22]. These studies indicated that the main function of MmuPV1 E8^E2 is preventing over replication as this initiates the productive replication phase in undifferentiated cells leading to their growth arrest. We now extended these findings to the high risk-HPV31, as HPV31 E8^E2 mt genomes are unable to be maintained as episomes in stable cell lines and are also impaired in their ability to induce immortalized keratinocyte cell lines [24,27]. Comparable to MmuPV1, HPV31 E8- and E8 KWK mt genomes induce viral late transcripts and E4 protein expression in normal human keratinocytes maintained under conditions favoring the growth of undifferentiated, basal-like cells.

E8^E2 proteins functionally interact with the multiprotein NCoR/SMRT co-repressor core complexes [16–18]. Remarkably, depletion of specific NCoR/SMRT complex components in cell lines stably maintaining HPV31 episomes results in E4 protein expression, viral late gene transcription, and formation of viral replication centers, markers for the start of the productive cycle. This indicates that the E8^E2 repressor limits genome amplification in the initial amplification phase after cell entry, but also during the maintenance replication phase to prevent vegetative genome amplification. The depletion of NCoR and SMRT, but not of HDAC3, induced genome amplification and E4 expression, consistent with previous studies demonstrating that HDAC3 does not significantly contribute to repression by E8^E2 proteins [16,18,21]. Interestingly, depletion of TBL1 and TBLR1 activated genome amplification and E4 expression as efficient – and in some assays even better - as the knockdown of NCoR and SMRT suggesting that TBL1/TBLR1 proteins are crucial for the repression by E8^E2. TBL1/TBLR1 proteins form dimers and tetramers and bind directly to NCoR/SMRT proteins and GPS2 and thus may act as a scaffold [47]. Furthermore, they interact with histones which may keep NCoR/SMRT complexes stably chromatin-associated [48]. Nevertheless, in contrast to the other complex components the contributions of TBL1/TBLR1 to repression are not well defined. The dependency on NCoR/SMRT and TBL1/TBLR1, but independency from HDAC3, is different from repression by thyroid hormone receptor and for the *SREBP1* and *BMAL1* genes in CIN612-9E cells, which suggests a novel repression mechanism, possibly specific for HPV [49].

It is generally accepted that the productive replication cycle of HPV requires keratinocyte differentiation. Current models suggest that, after re-entry into the cell cycle, the first step in suprabasal HPV-infected cells is the activation of PL by differentiation-activated transcription factors such as C/EBP-beta which not only drives expression of E4, but also of the E1 and E2 replication proteins, thereby inducing genome amplification [5–8]. In addition, the activation of the ATR and ATM DNA damage response pathways is required to facilitate vegetative genome amplification [3,9]. Nevertheless, 3D reconstruction and differentiation marker analysis indicate that ~50% of E4-expressing HPV31 E8^E2 mt keratinocytes are attached to the tissue culture plate and do not express the suprabasal keratinocyte marker KRT10 suggesting that they have basal-like keratinocyte properties. Consistent with this, ~ 90% of E4-positive cells induced by depletion of NCoR/SMRT complex components are attached to the culture plate and are KRT10-negative, whereas 84% of the E4-expressing cells in the control grow on top of other cells and ~25% are KRT10-positive consistent with differentiation being required for entering the productive replication cycle. The analysis of the transcript levels of different keratinocyte differentiation markers also provided no evidence that disruption of NCoR/SMRT-complexes in HPV31-positive cells induces differentiation. The observation that HPV31 E8^E2 mt genomes fail to establish stable keratinocyte cell lines maintaining episomes is also consistent with the idea that E8^E2 mt genomes induce genome amplification and E4 expression in undifferentiated keratinocytes which prevents them from dividing and forming colonies. Taken together, these data strongly suggest that the inactivation of E8^E2 enables genome amplification and E4 expression in undifferentiated keratinocytes. Interestingly, the number of E4-positive cells became similar between control and NCoR/SMRT-complex depleted cells upon differentiation in methyl-cellulose medium. This strongly suggests that the inactivation of E8^E2 is a key step in differentiating HPV31-positive keratinocytes to enable vegetative genome replication. Since *E8^E2* transcripts are expressed at similar or even higher levels in differentiated than in undifferentiated HPV-positive keratinocytes [27,50,51], it is more likely that the activity of E8^E2 is controlled at the post-transcriptional level which requires future studies.

Productive HPV replication takes place in a prolonged G2-phase and such cells do not enter mitosis but eventually exit the cell cycle [42,43]. Our previous study using MmuPV1 suggested that the expression of E4 protein is mainly responsible for a shift into G2 in undifferentiated cells [17]. Interestingly, neither the inactivation of E4 nor the additional inactivation of E5, which can also be expressed from the major PL *E1^E4, E5* transcript, enabled the establishment of HPV31 E8- episomes. This strongly suggests that late HPV31 protein expression is not responsible for the failure of E8- genomes to establish episomal cell lines. A major fraction of E4-positive cells induced by mutation of E8^E2 or depletion of NCoR/SMRT components display cytoplasmic cyclin B1 expression consistent with these cells being in the G2-phase. Furthermore, almost all E4-positive cells express γH2AX, which can be induced by E6, E7, and E1-mediated genome replication, consistent with activated ATM and ATR DNA damage response pathways. ATR activates CHK1 kinase, which is not only important for vegetative HPV31 genome replication, but also arrests cells in G2 via the inactivation of CDK1 [52,53]. In addition, damaged DNA can also be sensed by the cGAS/STING pathway and induce innate immunity pathways with antiviral activity [54]. Thus, it is likely that the activation of DNA damage response pathways by vegetative genome amplification and E6 and E7 expression is sufficient to prevent cell division and therefore the establishment of episomal E8- cell lines in the absence of E4 and E5. However, we also noted that the fraction of cytoplasmic cyclinB1-/E4-positive cells decreases upon extended culturing or differentiation suggesting that the siRNA-induced genome amplification and E4 expression is similar to differentiation-induced genome amplification and E4 expression.

In contrast to HPV31, HPV16 and beta-HPV49 E8- genomes can be stably maintained as episomes in keratinocytes [24–26]. HPV16 E8^E2 mt cell lines display higher levels of viral copies in undifferentiated keratinocytes, but E4 protein expression can only be detected in suprabasal differentiated layers in organotypic cultures [24,26]. This suggests that HPV16 requires a differentiation-dependent step in addition to the inactivation of E8^E2 to enter the productive replication cycle which should be addressed in the future.

In summary, or data support the idea that targeting the E8^E2-NCoR/SMRT complex interaction to induce genome amplification and E4 expression in undifferentiated cells could be an antiviral strategy for some HPV infections.

## Materials and methods

### Ethics statement

NHK were isolated from human foreskin upon informed consent of participants or their legal guardian. The procedure was approved by the ethics committee of the medical faculty of the University Tuebingen (6199/2018BO2), and done according to the principles of the Declaration of Helsinki.

### Recombinant plasmids

The plasmid encoding human PGK1 has been previously described [25]. Plasmids pUC57 HPV31 E6* (HPV31 nt. 108-210/ 413-420) and pUC57 HPV31 E1^E4^L1 (HPV31 nt. 86-877/3295-3578/ 5552-5688) used as copy number standards were obtained by gene synthesis (GenScript). The cloned HPV31 wt, E8-, and E8 KWK mt genomes have been previously described [27,29]. Previously described E4M9 and mE5-1 mutations [31,33] were generated by PCR and introduced by standard cloning techniques into the respective genomes giving rise to E4M9, E4M9/E8-, E4M9/mE5-1, and E4M9/mE5-1/E8- genomes. To obtain plasmid pSG5 flag-TBL1, the cloned TBL1 cDNA was amplified from Horizon MHS6278-202833338 clone id: 6378852 by PCR and inserted with an N-terminal flag tag into pSG5. pSG-TBLR1 was generated by inserting a PCR-amplified TBLR1 cDNA into pSG5 and used to validate TBLR1-specific qPCR primers. To generate a pIRES-TBLR1 co-flag expression plasmid, a commercially-obtained, codon-optimized version of human TBLR1 was cloned with a C-terminal flag tag into pIRES-puro (Clontech). All plasmids were verified by sequencing of the complete plasmid.

### Cell culture

NHK were maintained in KSFM media (Thermo Fisher) HPV31 wt and mt cell lines were obtained by transfection of re-circularized genomes as previously described [28]. HK-HPV31, HK-HPV31 E6/E7 [27,55], and CIN612-9E cell lines were co-cultured with Mitomycin C-treated NIH3T3 J2 mouse fibroblasts in E-medium with supplements as previously described [28]. HeLa cells were cultured in DMEM/10% FBS and antibiotics. 300.000 HeLa cells were seeded in 6 well plates for immunoblot analysis. NHK were seeded at a density of 10.000 cells/cm^2^ for transfection experiments. In the case of CIN612-9E or HK-HPV31 wt cell lines, 17.000-23.000 cells/cm^2^ were seeded for short-term experiments and 10.000 cells/cm^2^ for long-term experiments.

### Methylcellulose differentiation

CIN612-9E cells were seeded into cell culture plates, transfected with siRNA after 24 h, differentiated in complete E-media without EGF, supplemented with 1.5% (w/v) methylcellulose (Sigma M-0512) as described [36]. Cells were harvested 48 h later and processed for RNA and IF analysis.

### Plasmid and siRNA transfection

For the analysis of transiently replicating HPV31 genomes, NHK were seeded and transfected 24 h later with re-circularized HPV31 genomes (1 and 0.5 µg in 6- and 12-well plates, respectively) with 2.5 µl/µg DNA FuGeneHD (Thermo Fisher). To analyze overexpressed TBL1 and TBLR1 proteins by immunoblot, HeLa cells were transfected with 660 ng of the corresponding expression plasmids and FuGeneHD (2.5µl/µg DNA). For knock-down experiments, cells were transfected with 80/40/16 pmol siRNA and 7/3.5/1.4 µl of RNAiMAX transfection reagent (Thermo Fisher) in 6-well plates and collagen coated glass bottom dishes/12-well plates/8-well chamber slides with the following siRNAs: siControl (siAllstars negative control; #1027281; Qiagen), siHDAC3 (hsHDAC3, Dharmacon, #L-003496-00), siNCoR1 (hsNCOR1, Dharmacon, #L-003518-00), siSMRT (hsSMRT, Dharmacon, #L-020145-01), siTBL1 (hsTBL1X, Dharmacon, #L-012152-00), or siTBLR1 (hsTBL1XR1, Dharmacon, #L-012927-00).

### Immunoblot analysis

Cell extracts were prepared by lysis in RIPA buffer (1% (v/v) IGEPAL CA-630; 1% (v/v) sodium deoxycholate; 0.1% (v/v) SDS; 150 mM sodium chloride; 10 mM sodium phosphate (pH = 7.2); 2 mM EDTA; 50 mM sodium fluoride; protease and phosphatase inhibitors), mixed with Laemmli-buffer (Carl Roth, ROTI Load 1 (reducing), #K929), heated to 95 °C for 5 min and then sonified. Proteins were blotted onto 0.22 μm nitrocellulose membranes (Amersham). Antibodies detecting HSP90 (4F10, mouse, Santa Cruz, #sc69703, 1:2000) or GAPDH (6C5, mouse, Santa Cruz, #sc32233, 1:1000) served as loading controls. The following primary antibodies were used: CCNA2 (E6D1J, rabbit, CellSignaling, #67955, 1:1000), CCNB1 (D5C10, XP, rabbit, CellSignaling, #12231, 1:1000), Flag-Tag (DYKDDDDK; D6W5B, rabbit, CellSignaling, #14793, 1:1000), Flag-Tag (DYKDDDDK, mouse, CellSignaling, #8146, 1:1000), TBL1 (mouse, Santa Cruz, #sc137006, 1:1000) and TBL1XR1/TBLR (rabbit, CellSignaling, #74499, 1:1000). Primary antibodies were incubated o/n at 4 °C. Secondary antibodies (goat anti-rabbit-IRDye680, LI-COR Biotechnology, 926-68071; anti-mouse-IRDye800, LI-COR Biotechnology, 926-32210) were diluted 1:15000 in PBS and incubated for 1 h at room temperature. Bound antibodies were detected using an Odyssey Fc imager (LI-COR Biotechnology).

### Nucleic acid isolation and quantitative PCR

RNA was isolated using QIAshredder (Qiagen) and the RNeasy Mini Kit (Qiagen) according to the manufacturer´s recommendations. CDNA was synthesized using the QuantiTect RT Kit (Qiagen) and 50 ng cDNA was used per reaction using a LightCycler480 II (Roche) and the LightCycler480 SYBR green Master mix I (Roche).

Primers for *HDAC3*, *PGK1,* HPV31 E6*, HPV31 *E1^E4,* and HPV31 *E4^L1* transcripts have been previously described [21,26,56]. *TBLR1* was detected with a commercially available assay (Hs_TBL1XR1_1_SG, Qiagen, GeneGlobe ID: QT00197323, Cat. No.: 249900), *TBL1* was detected with the forward primer AGGGACACACAAACGAGGTC and the reverse primer AATGTCATGTCATCCGAGCA. NCoR1 was detected using the following primers (F: GAAAGACTGCCAACAGTCAGG; R: CATCGAGAGGTCTCCACAGG) and SMRT was detected using the following primers (F: GCACGAGGTGTCAGAGATCA, R: GAACTTCTCCCGGAAGGTCT). Total genomic DNA was isolated using the DNeasy Blood & Tissue Kit (Qiagen, #69504). The exonucleaseV-resistance assay was adapted from [32] with minor modifications: total cellular DNA (100 ng) was incubated in the presence or absence of 5 U exonuclease V (NEB M0345S) in 1 × NEBuffer 4 supplemented with 1 mM ATP for 60 min at 37°C. Then, the enzyme was inactivated for 10 min at 95°C. Finally, 10 ng input DNA was measured by qPCR using the following primers for HPV31 (F: CTGTTGTGGAAGGGCAAGTT, R: TCCCAGCAAAGGATATTTCG), *FLNB* (F: GGAAGGTCCGTGCGAGG, R: GAGACGGAGCAGGTCCCA), or the mitochondrial genome (F: GAGGAACAGCTCTTTGGACA, R: CAATTGGGTGTGAGGAGTTC) and standard curves. Then, the fraction of exonucleaseV-resistant HPV31, mitochondrial genomes, and the cellular *FLNB* gene were calculated.

### RNA-sequencing analysis

CIN612-9E cells (3 replicates) were transfected with siControl, siHDAC3, siNCoR/SMRT, or siTBL1/TBLR1 and RNA was isolated 48h post transfection. Total RNA was subjected to strand-specific library preparation using the CORALL mRNA-Seq V2 Kit (Lexogen) according to the manufacturer’s instructions. Libraries were quality-controlled on a TapeStation D1000 Assay (Agilent) and sequenced on an Illumina NextSeq 2000 using the P3 Reagents (100 Cycles) kit (product number: 20040559) in paired-end mode (2 × 57 bp). All samples passed quality control assessed by FastQC.

Strand-specific gene counts were quantified using STAR against the human GRCh38 genome assembly in combination with the ENSEMBL/GENCODE gene annotation (version 105). Differential gene expression analysis was performed using DESeq2, with differentially expressed genes defined by FDR (Benjamini-Hochberg-adjusted p-value) < 0.1 and |log₂ fold change| > 1.

### Immunofluorescence

For IF analysis, NHK, CIN612-9E, and HK-HPV31 wt cells were seeded onto MatTek collagen-coated 35 mm glass bottom dishes (MatTek, P35GCol-1.5-14-C) and transfected 24 h later. Cells were fixed with 4% PFA for 15 min at RT. Methylcellulose-differentiated cells and the corresponding control cells were counted after removal of the methylcellulose medium by centrifugation in PBS. Cells were fixed in 4% PFA for 10 min at RT, centrifuged (300 g, 5 min), washed once with PBS, and then resuspended in PBS. Then, 4x10^4^ cells per sample were attached to glass slides by centrifugation (Cytospin3, Shandon) using the corresponding slide holders and then quickly dried at RT. Cells were then incubated for 1 h at RT with blocking buffer (1x PBS, 5% (v/v) normal goat and donkey serum, 0.3% (v/v) Triton-X 100). Primary antibodies were diluted in PBS, 1% (w/v) BSA fraction V, 0.3% (v/v) Triton-X 100 and incubated overnight at 4°C using the following antibodies: Pan-HR-E4 FH1.1 (mouse monoclonal, PMID: 25953390; 1:100), KRT10 (rabbit monoclonal, abcam, #ab76318, 1:150), CCNB1 (D5C10) XP (rabbit monoclonal, CellSignaling #12231, 1:500), CCNA2 (E6D1J) (rabbit monoclonal, Cell Signaling #67955 1:500), p53 (7F5) (rabbit monoclonal, CellSignaling #2527 1:1000), p21 Waf1/Cip1 (12D1) (rabbit monoclonal CellSignaling #2947 1:400), γH2AX [Phospho Ser139-Histone H2A.X, (20E3), rabbit monoclonal, CellSignaling #9718, 1:400). Primary antibodies were detected with the following secondary antibodies: Alexa Fluor 488 goat anti-mouse IgG (Invitrogen by Thermo Fisher Scientific, A11029, 1:1000), Alexa Fluor 555 goat anti-mouse IgG (Invitrogen by Thermo Fisher Scientific, A31570, 1:1000), Alexa Fluor 488 donkey anti-rabbit IgG (Invitrogen by Thermo Fisher, A21206, 1:1000), Alexa Fluor 647 donkey anti-rabbit (Invitrogen by Thermo Fisher, A31573, 1:1000) for 1 h at RT. Glass bottom dishes, were finally washed with water, overlaid with water and analyzed with a Zeiss Axio Observer Z1 fluorescence microscope using an 20x or 63x objective and the appropriate filter sets in combination with an Apotome2 and the Zeiss Zen 3.9 software. For quantification, at least 10 random images were taken per experiment and DAPI-stained cell nuclei were automatically counted with the Zeiss Zen 3.9 software, while other signals (E4, CCNB1, CCNA2, p53, p21, γH2AX) were counted manually after applying a threshold. For the analysis of the position of cells relative to other cells and the tissue culture plate surface, z-stacks were recorded (63x objective) and 3D reconstructions were analyzed manually using Zeiss Zen 3.9 software.

### DNA-FISH

HPV31 genomes were detected by FISH using the RNAscope Multiplex Fluorescent Reagent Kit v2 (Bio-Techne, #323100), RNAscope H_2_O_2_ and Protease reagents (#322381) with TSA fluorophore (TSA-Vivid 570, #323272) diluted in RNAscope Multiplex TSA Buffer (#322809) and the 10 zz pair C1-labelled RNAscope Probe - HPV31 (# 311551) and subsequent IF staining. Detection was carried out accordingly to the manufacturer’s instructions and the previously published modifications to detect viral DNA [57] . In short, 3x10^4^ CIN612-9E cells were seeded onto an 8 well chamber polystyrol vessel tissue culture treated glass slide (Falcon #354118), transfected 24 h later and fixed additional 48 h later with 4% PFA for 15 min. Cells were permeabilized with PBS-T (0.1%) for 10 min, treated for 10 min with H_2_O_2,_ and then 10 min with diluted proteaseIII. Between all steps, the slides were washed with PBS. Samples were incubated with RNAse (25 µg/ml) for 30 min at 37 °C slides and then denatured in 70% formamide for 4 min at 82°C. After washing, slides were incubated for 15 min at 60 °C with prewarmed probe, then put immediately on 40°C overnight. Amplification steps 1, 2 and 3 were done at 40 °C, then the signal was developed with HRP-C1 and labelled with TSA-Vivid dye 570 (1:1.500). After stopping the reaction with HRP-blocker, IF staining was carried out. Samples were incubated with blocking reagent (PBS, 10% normal serum, 1% BSA, 0.3% Triton X-100) for 1 h at RT. The primary antibody was incubated for 3 h at RT (FH1.1 pan-HR-E4, 1:100), and then the secondary antibody was added for 1 h (Alexa Fluor 488 goat anti-mouse IgG, Invitrogen by Thermo Fisher Scientific, A11029, 1:1000). Cell nuclei were stained with DAPI for 1 min, washed and mounted (25% [v/v] glycerol, 10% [w/v] Mowiol 488, 100 mM Tris-HCl pH 8). Slides were imaged with a Zeiss Axio Observer Z1 fluorescence microscope using an 63x objective and the appropriate filter sets in combination with the Apotome2 and Zeiss Zen software (version 3.9).

## Supporting information

S1 Fig (A) QPCR analysis of knockdown efficiency of siRNA-transfection of CIN612-9E cells targeting *TBL1*, *TBLR1* or both. Values were normalized to PGK1. Analysis was done by a mixed effects analysis with Dunnett’s multiple comparisons test (n = 3–8; *p = 0.05; **p = 0.01, **** p < 0.0001). Error bars indicate the SEM. (B) Western Blot analysis of human flag-tagged TBL1 and TBLR1 protein overexpressed in HeLa cells. The left membrane was stained with mouse anti-TBL1 antibody (Santa Cruz, #sc137006, 1:1000), which recognizes both TBL1 and TBLR1, and with anti-flag-tag rabbit-antibody (CellSignaling, #14793, 1:1000) as a control. On the right the same samples were stained with rabbit-antibody TBL1XR1/TBLR (CellSignaling, #74499, 1:1000), which only recognizes TBLR1, and anti-flag-tag mouse-antibody (CellSignaling, #8146, 1:1000) as a control. Shown below are the single channels in black and white. (C) Western Blot analysis of CIN612-9E cells 48h post transfection with transfected with siRNA against TBL1, TBLR1 or both. Anti-GAPDH was used as a loading control. The left membrane was stained with mouse anti-TBL1 antibody (Santa Cruz, #sc137006, 1:1000), which recognizes both TBL1 and TBLR1. On the right, the same samples were stained with rabbit-antibody TBL1XR1/TBLR (CellSignaling, #74499, 1:1000), which only recognizes TBLR1. Relative expression levels relative to siControl and normalized to GAPDH expression are indicated below. (D) QPCR analysis of spliced viral *E1^E4* transcript 48 h after transfection of CIN612-9E cells with siRNA against *TBL1*, *TBLR1* or a combination of both. Values were normalized to *PGK1* and are shown relative to siControl. Statistical significance was determined using a mixed effects analysis with the Dunnett’s multiple comparisons test (n = 3–9, **p = 0.01). Error bars indicate the SEM.
(PNG)

S2 Fig QPCR measurements of siRNA-knockdown efficiencies of the NCoR/SMRT co-repressor complex in HK-HPV31 (n = 6) and CIN612-9E (n = 7) cell lines. Values were normalized to *PGK1* and are shown relative to siControl. A mixed-effects analysis with Šídák’s multiple comparisons test was used to determine significance (n = 4–7, *p = 0.05; **p = 0.01; *** p = 0.001, **** p < 0.0001). Error bars indicate the SEM.
(PNG)

S3 Fig 3D image analysis of the fraction of E4-positive cells in HK-HPV31 cells 48h after siRNA-transfection. Representative images are shown on the right and the bar graph on the left shows the quantification of E4-positive cells and their relative position. Data was analyzed by two-way ANOVA with Tukey’s multiple comparisons test (n = 7–8; **** p < 0.0001). Error bars indicate the SEM.
(PNG)

S4 Fig Analysis and exemplary images (right) of HPV31 wt expressing keratinocyte-based cell lines 48 h after siRNA-transfection. The fractions of E4 + / cyclin B1+ (CCNB1) cells are shown on the left. The statistical analysis was done by ordinary one-way ANOVA with Dunnett’s multiple comparisons test (n = 3, **** p < 0.0001). On the right, localization of these cells relative to the tissue culture surface are shown (basal/ “suprabasal”). Statistical analysis was done by a mixed-effects analysis with Tukey’s multiple comparisons test (n = 2–3, *** p = 0.001, **** p < 0.0001). Representative images are shown on the right. Magnification is 630x, the scale bar is 10µm and DNA was stained with DAPI.
(PNG)

S5 Fig Representative western blot of HK-HPV31 E6/E7-expressing keratinocytes 48h after siRNA-transfection (*Control, TBL1/TBLR1*) stained for CCNB1, CCNA2, TBL1/TBLR1 (Santa Cruz, #sc137006, 1:1000) and HSP90 as a loading control. (B) Quantification of Western blot signals from (A). Signals from three independent experiments in HK-HPV31 E6/E7-expresssing cell lines were normalized to HSP90 and set relative to the siControl. Statistical analysis was done by two-way ANOVA with a Dunnett’s multiple comparisons test. Error bars indicate the SEM (n = 3, *p = 0.05).
(PNG)

S6 Fig Knockdown efficiencies in CIN612-9E cells, 2/4/ 6 d after siRNA transfection, each relative to siControl, normalized to *PGK1.* Kruskal-Wallis tests were used to determine statistical significance, with the Dunn’s multiple comparisons test (n = 2, **p = 0.01; *** p = 0.001, **** p < 0.0001). Error bars indicate the SEM.
(PNG)

S7 Fig QPCR analysis of knockdown efficiencies in CIN612-9E cells 48 h p.t. with siRNAs against different NCoR/SMRT complex components, and suspension in methylcellulose-media for additional 48 h in comparison to cells grown in monolayer. Values were normalized to *PGK1* and set relative to siControl in monolayer. Statistical significance was determined using a mixed-effects model with Tukey’s multiple comparisons test (n = 4–5 experiments, *p = 0.05; **** p < 0.0001). Error bars indicate the SEM.
(PNG)
