# Supplementary figures and images for: Inhibition of high risk HPV31 E8^E2 repressor activity enables differentiation-independent genome amplification and E4 expression
Source: PLoS Pathog. 2026 Jun 8;22(6):e1014330. doi: 10.1371/journal.ppat.1014330 (PMC13268182; doi:10.1371/journal.ppat.1014330)

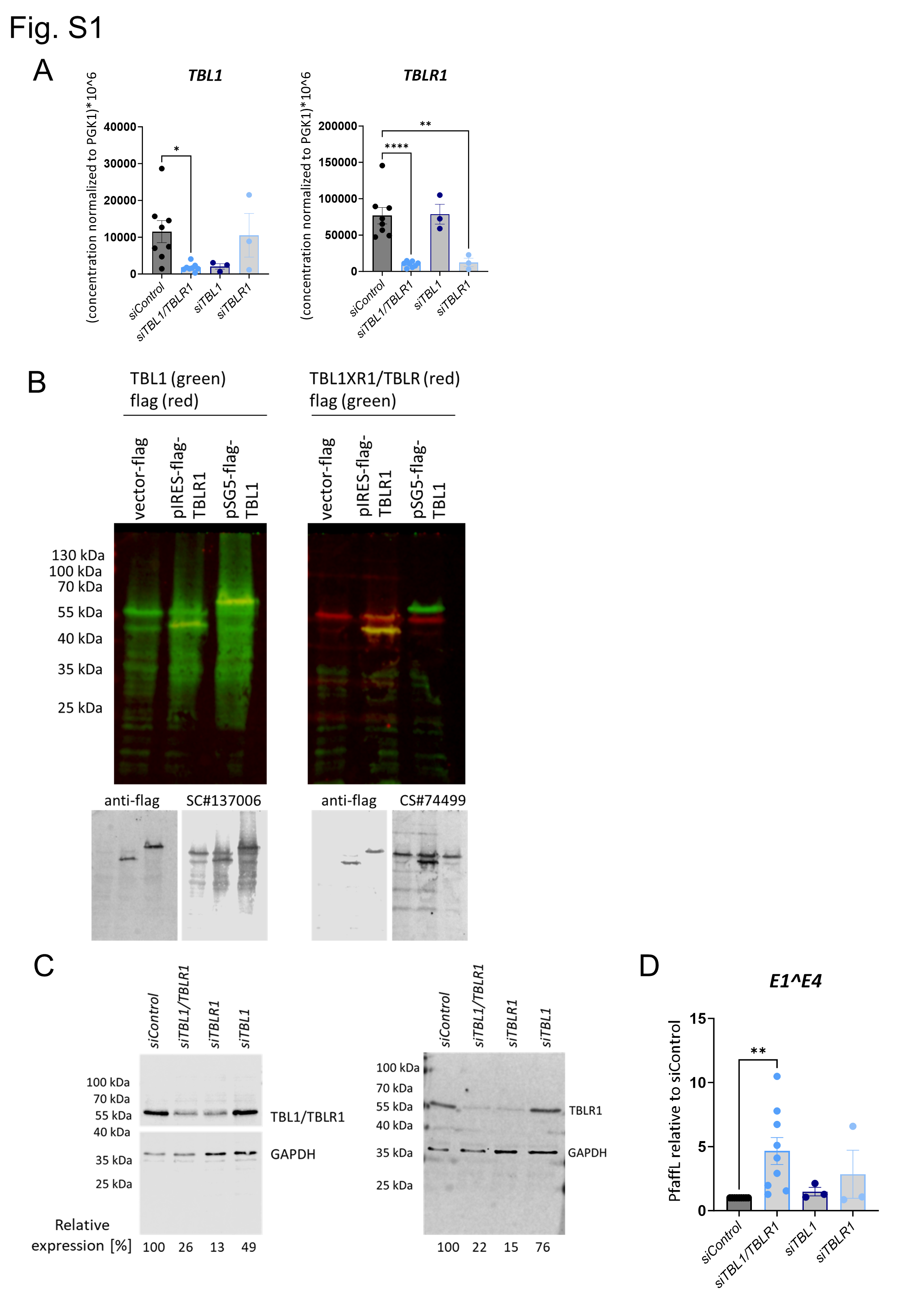

Supplement: S1 Fig — Values were normalized to PGK1. Analysis was done by a mixed effects analysis with Dunnett’s multiple comparisons test (n = 3–8; *p = 0.05; **p = 0.01, **** p < 0.0001). Error bars indicate the SEM. (B) Western Blot analysis of human flag-tagged TBL1 and TBLR1 protein overexpressed in HeLa cells. The left membrane was stained with mouse anti-TBL1 antibody (Santa Cruz, #sc137006, 1:1000), which recognizes both TBL1 and TBLR1, and with anti-flag-tag rabbit-antibody (CellSignaling, #14793, 1:1000) as a control. On the right the same samples were stained with rabbit-antibody TBL1XR1/TBLR (CellSignaling, #74499, 1:1000), which only recognizes TBLR1, and anti-flag-tag mouse-antibody (CellSignaling, #8146, 1:1000) as a control. Shown below are the single channels in black and white. (C) Western Blot analysis of CIN612-9E cells 48h post transfection with transfected with siRNA against TBL1, TBLR1 or both. Anti-GAPDH was used as a loading control. The left membrane was stained with mouse anti-TBL1 antibody (Santa Cruz, #sc137006, 1:1000), which recognizes both TBL1 and TBLR1. On the right, the same samples were stained with rabbit-antibody TBL1XR1/TBLR (CellSignaling, #74499, 1:1000), which only recognizes TBLR1. Relative expression levels relative to siControl and normalized to GAPDH expression are indicated below. (D) QPCR analysis of spliced viral E1^E4 transcript 48 h after transfection of CIN612-9E cells with siRNA against TBL1, TBLR1 or a combination of both. Values were normalized to PGK1 and are shown relative to siControl. Statistical significance was determined using a mixed effects analysis with the Dunnett’s multiple comparisons test (n = 3–9, **p = 0.01). Error bars indicate the SEM. (PNG) [file ppat.1014330.s001.PNG]

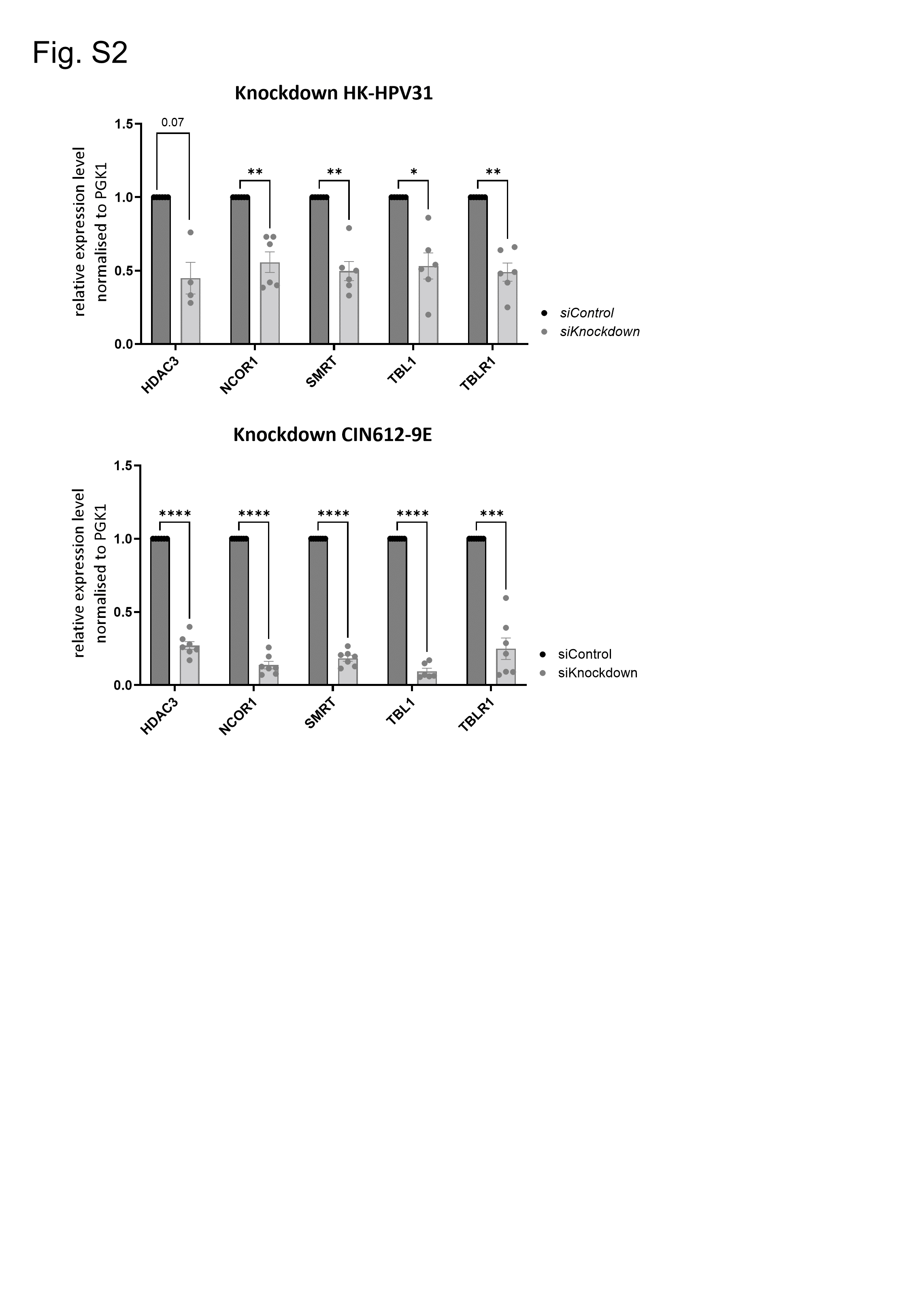

Supplement: S2 Fig — Values were normalized to PGK1 and are shown relative to siControl. A mixed-effects analysis with Šídák’s multiple comparisons test was used to determine significance (n = 4–7, *p = 0.05; **p = 0.01; *** p = 0.001, **** p < 0.0001). Error bars indicate the SEM. (PNG) [file ppat.1014330.s002.PNG]

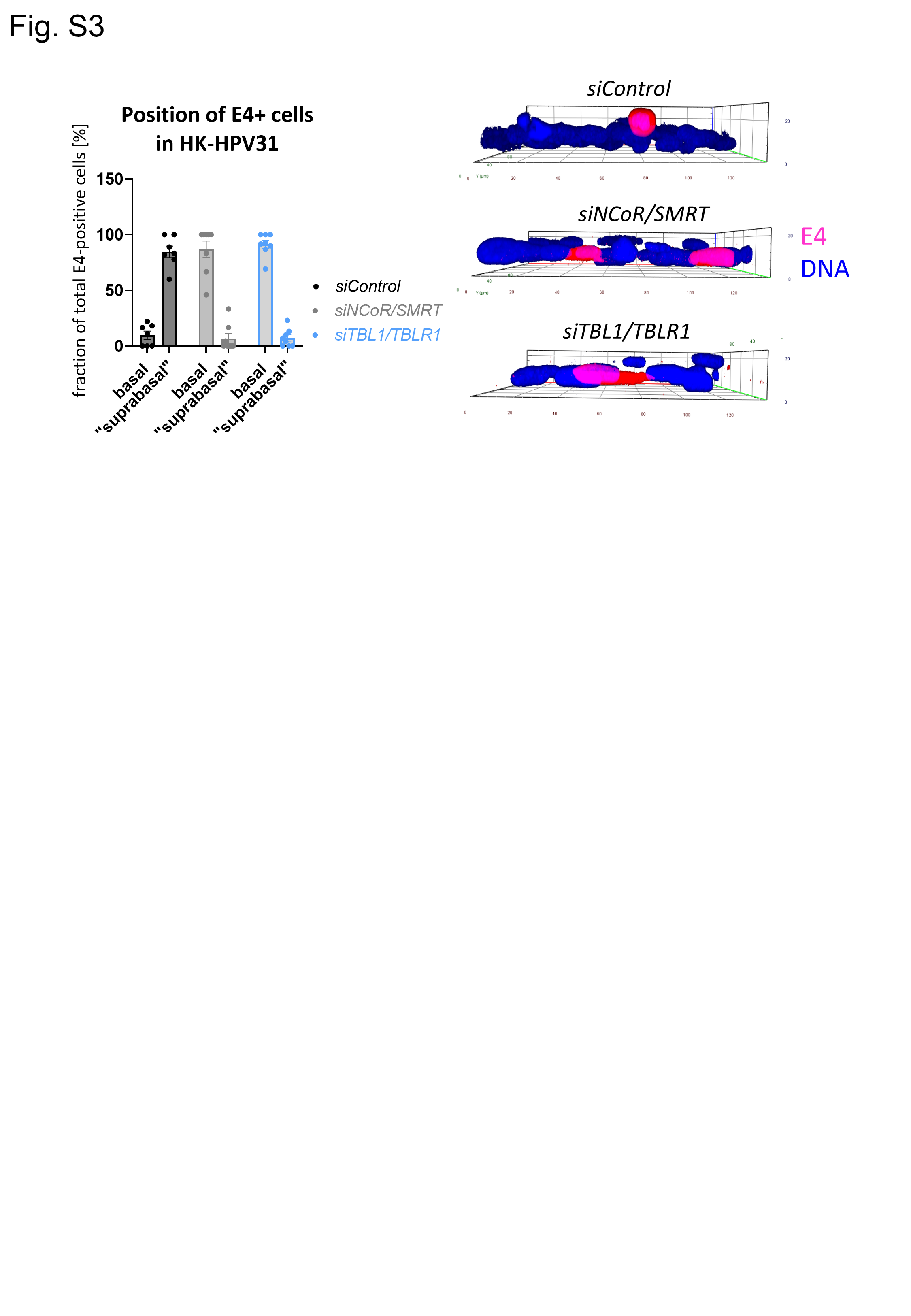

Supplement: S3 Fig — Representative images are shown on the right and the bar graph on the left shows the quantification of E4-positive cells and their relative position. Data was analyzed by two-way ANOVA with Tukey’s multiple comparisons test (n = 7–8; **** p < 0.0001). Error bars indicate the SEM. (PNG) [file ppat.1014330.s003.PNG]

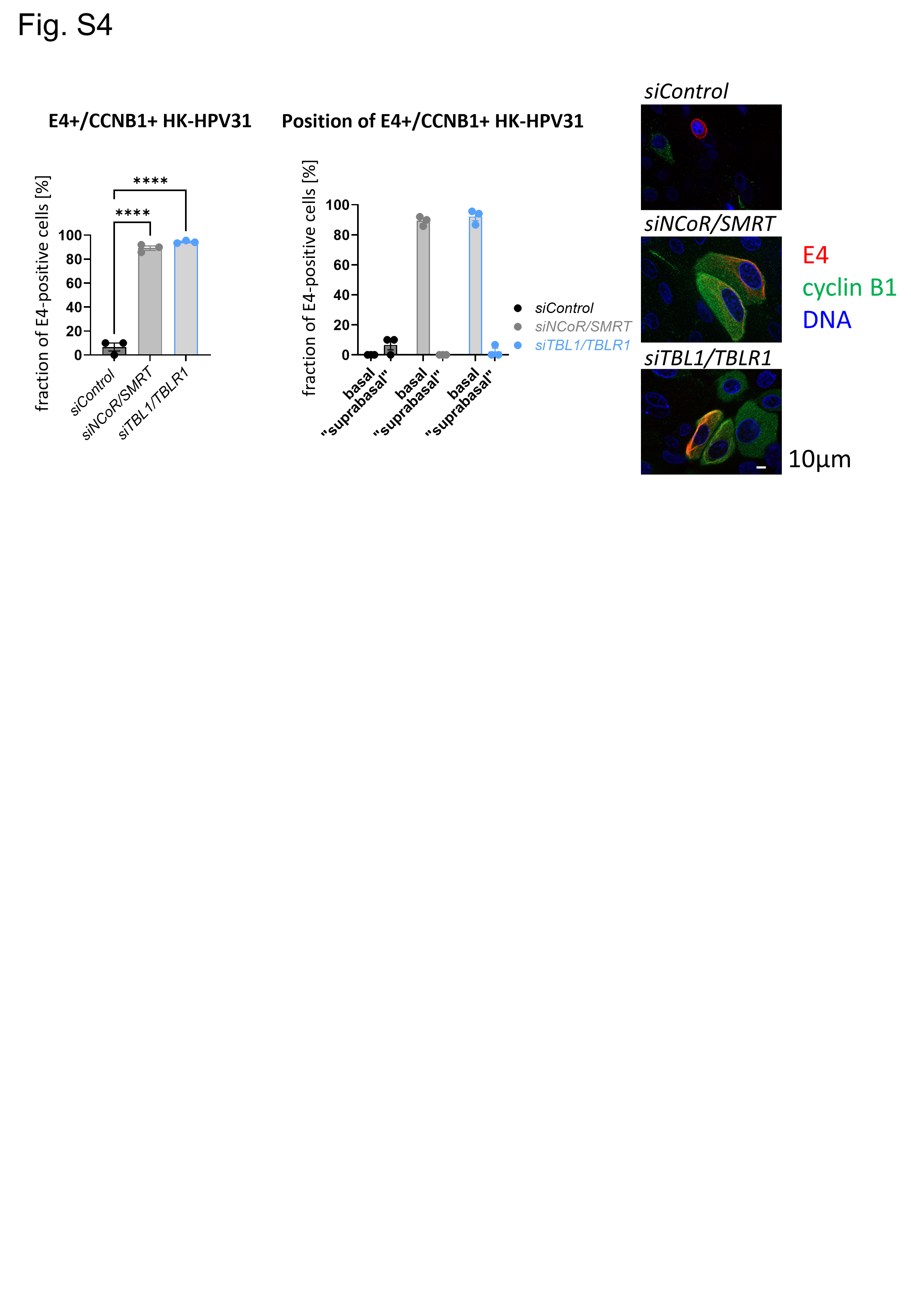

Supplement: S4 Fig — The fractions of E4 + / cyclin B1+ (CCNB1) cells are shown on the left. The statistical analysis was done by ordinary one-way ANOVA with Dunnett’s multiple comparisons test (n = 3, **** p < 0.0001). On the right, localization of these cells relative to the tissue culture surface are shown (basal/ “suprabasal”). Statistical analysis was done by a mixed-effects analysis with Tukey’s multiple comparisons test (n = 2–3, *** p = 0.001, **** p < 0.0001). Representative images are shown on the right. Magnification is 630x, the scale bar is 10µm and DNA was stained with DAPI. (PNG) [file ppat.1014330.s004.PNG]

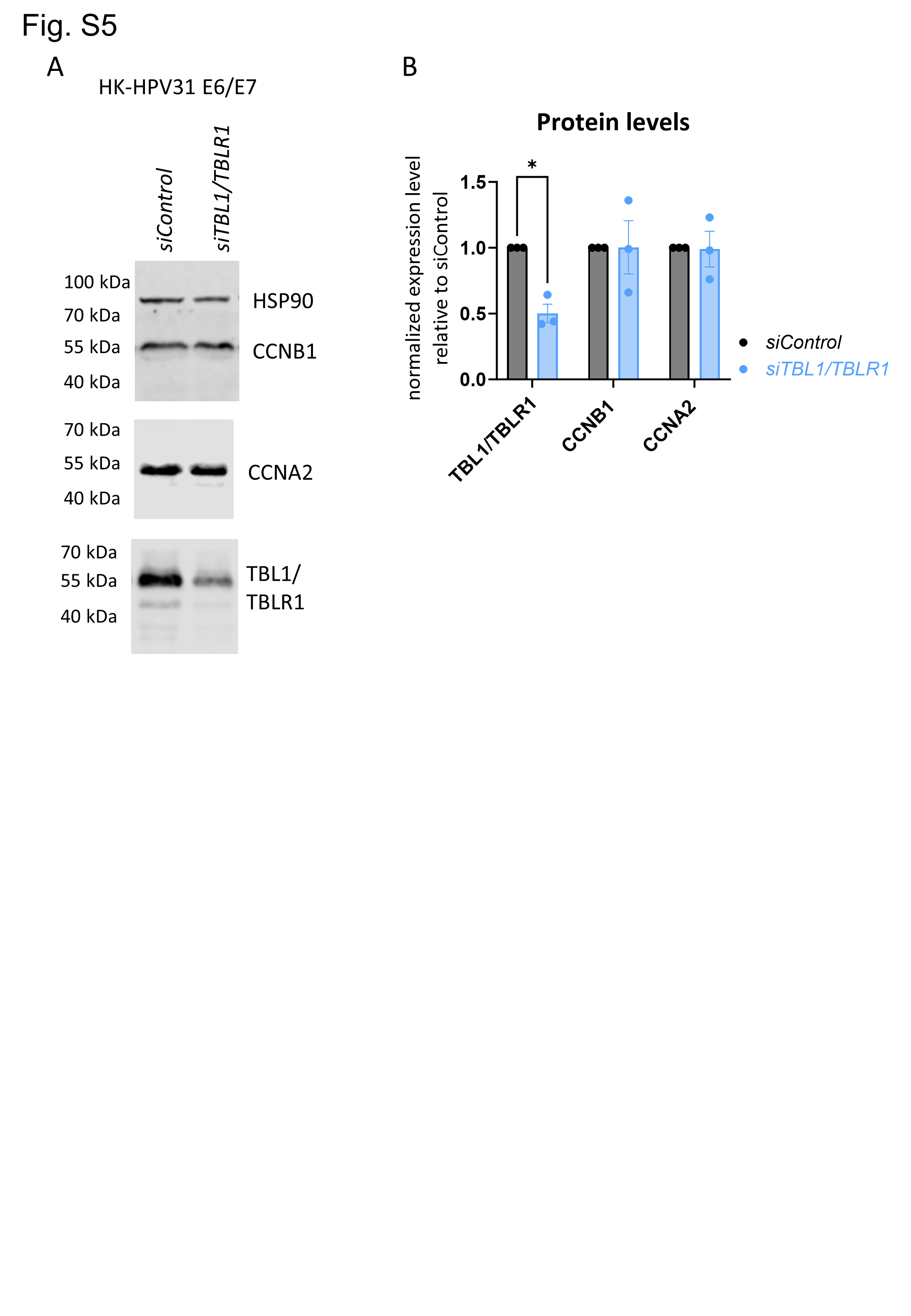

Supplement: S5 Fig — (B) Quantification of Western blot signals from (A). Signals from three independent experiments in HK-HPV31 E6/E7-expresssing cell lines were normalized to HSP90 and set relative to the siControl. Statistical analysis was done by two-way ANOVA with a Dunnett’s multiple comparisons test. Error bars indicate the SEM (n = 3, *p = 0.05). (PNG) [file ppat.1014330.s005.PNG]

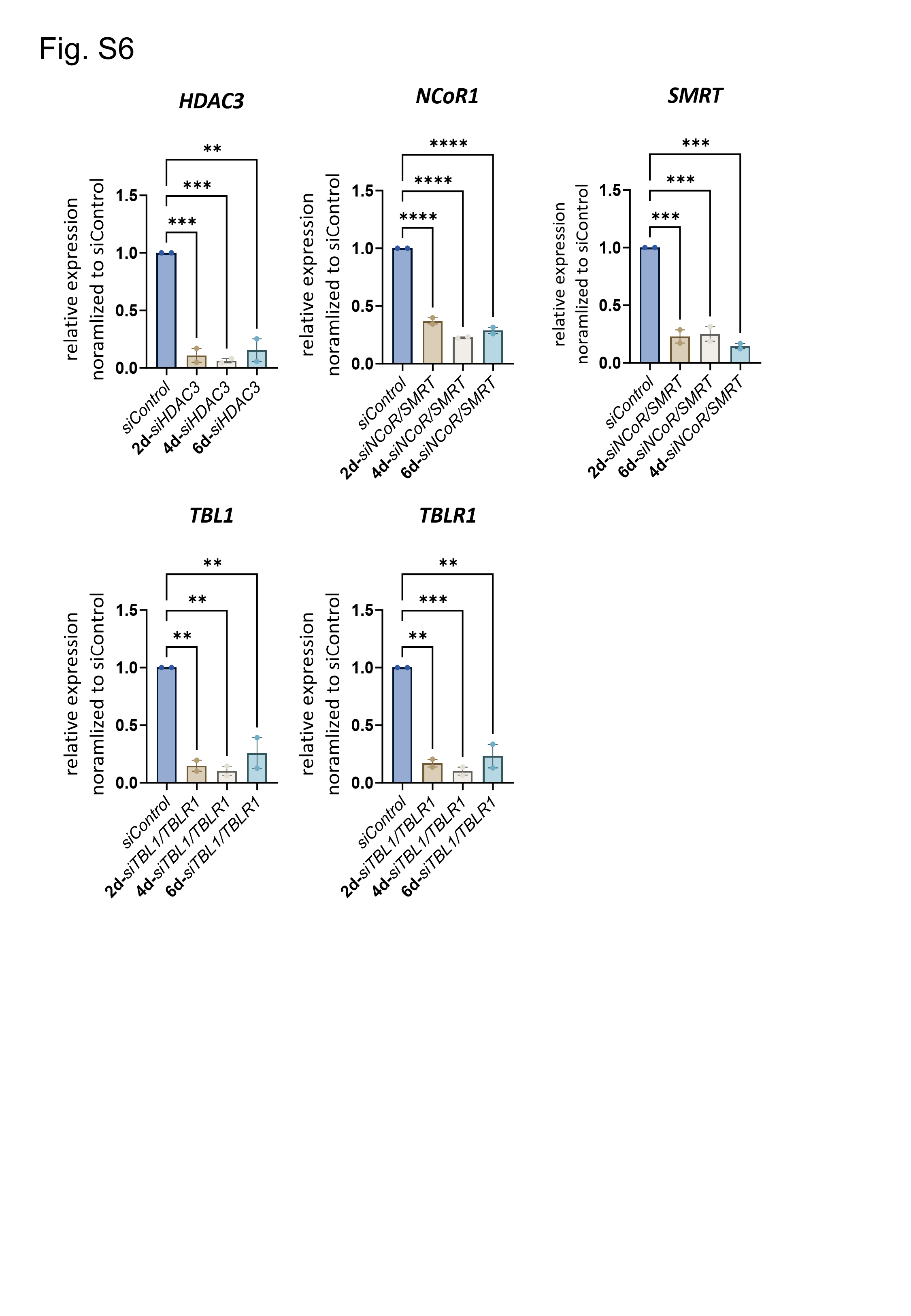

Supplement: S6 Fig — Kruskal-Wallis tests were used to determine statistical significance, with the Dunn’s multiple comparisons test (n = 2, **p = 0.01; *** p = 0.001, **** p < 0.0001). Error bars indicate the SEM. (PNG) [file ppat.1014330.s006.PNG]

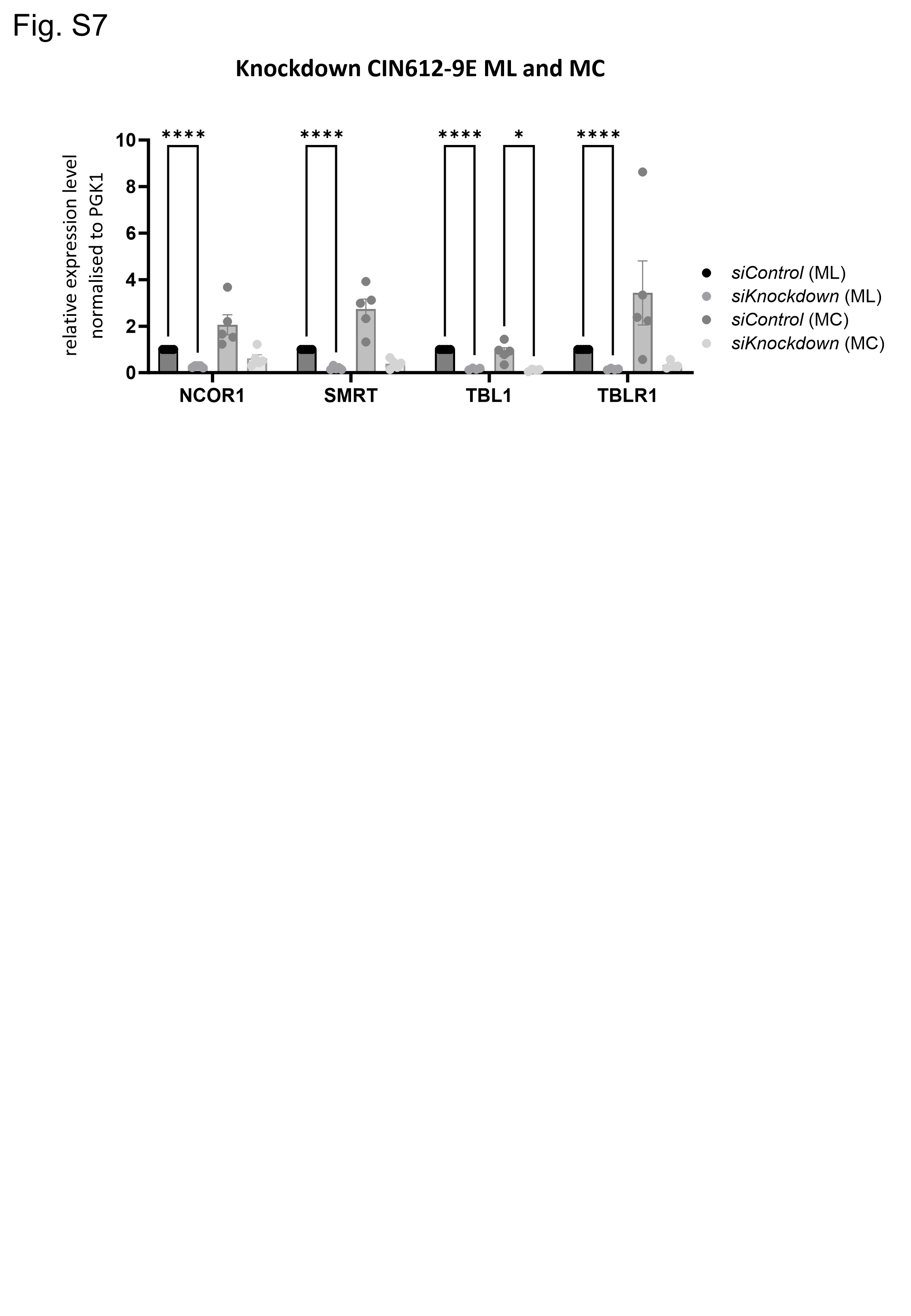

Supplement: S7 Fig — Values were normalized to PGK1 and set relative to siControl in monolayer. Statistical significance was determined using a mixed-effects model with Tukey’s multiple comparisons test (n = 4–5 experiments, *p = 0.05; **** p < 0.0001). Error bars indicate the SEM. (PNG) [file ppat.1014330.s007.PNG]
